# A Machine-Learning-Driven Pathophysiology-Based New Approach Method for the Dose-Dependent Assessment of Hazardous Chemical Mixtures and Experimental Validations

**DOI:** 10.3390/toxics12070481

**Published:** 2024-06-30

**Authors:** Sarita Limbu, Eric Glasgow, Tessa Block, Sivanesan Dakshanamurthy

**Affiliations:** Lombardi Comprehensive Cancer Center, Georgetown University Medical Center, 3700 O St. NW, Washington, DC 20057, USA

**Keywords:** environmental chemicals toxicity, PFAS, new approach methodology, machine learning, pathophysiology, dose-dependent toxicity, chemical-mixture interaction effect

## Abstract

Environmental chemicals, such as PFAS, exist as mixtures and are frequently encountered at varying concentrations, which can lead to serious health effects, such as cancer. Therefore, understanding the dose-dependent toxicity of chemical mixtures is essential for health risk assessment. However, comprehensive methods to assess toxicity and identify the mechanisms of these harmful mixtures are currently absent. In this study, the dose-dependent toxicity assessments of chemical mixtures are performed in three methodologically distinct phases. In the first phase, we evaluated our machine-learning method (AI-HNN) and pathophysiology method (CPTM) for predicting toxicity. In the second phase, we integrated AI-HNN and CPTM to establish a comprehensive new approach method (NAM) framework called AI-CPTM that is targeted at refining prediction accuracy and providing a comprehensive understanding of toxicity mechanisms. The third phase involved experimental validations of the AI-CPTM predictions. Initially, we developed binary, multiclass classification, and regression models to predict binary, categorical toxicity, and toxic potencies using nearly a thousand experimental mixtures. This empirical dataset was expanded with assumption-based virtual mixtures, compensating for the lack of experimental data and broadening the scope of the dataset. For comparison, we also developed machine-learning models based on RF, Bagging, AdaBoost, SVR, GB, KR, DT, KN, and Consensus methods. The AI-HNN achieved overall accuracies of over 80%, with the AUC exceeding 90%. In the final phase, we demonstrated the superior performance and predictive capability of AI-CPTM, including for PFAS mixtures and their interaction effects, through rigorous literature and statistical validations, along with experimental dose-response zebrafish-embryo toxicity assays. Overall, the AI-CPTM approach significantly improves upon the limitations of standalone AI models, showing extensive enhancements in identifying toxic chemicals and mixtures and their mechanisms. This study is the first to develop a hybrid NAM that integrates AI with a pathophysiology method to comprehensively predict chemical-mixture toxicity, carcinogenicity, and mechanisms.

## 1. Introduction

Humans are coexposed to various chemicals present in the environment. These environmental chemicals mainly exist as mixtures of diverse individual chemicals. The toxicity of these mixtures is significantly influenced by the concentration levels of their individual components. While the toxicity profiles of single chemicals have been widely studied and reported, there is a substantial gap in the comprehensive data regarding the toxicity of chemical mixtures. This lack of experimental toxicity data on chemical mixtures is due to several associated difficulties, including high costs, time-consuming processes, and ethical issues related to the use of animals in toxicological testing [1]. The existence of a large number of potential combinations and concentration ratios of chemical constituents further augments the practical challenges of conducting experimental evaluations on chemical mixtures [2]. As a result, there is a pressing need for alternative methods that can predict the toxicity of chemical mixtures without reliance on traditional experimental methods.

Computational toxicology offers a promising solution by employing mathematical and computer-based models to predict the effects of chemical exposures. These methods use existing data and predictive algorithms to evaluate potential health risks posed by chemical mixtures, thus circumventing some of the traditional challenges faced in experimental toxicity assessments [3]. Computational approaches are particularly valuable, as they can handle complex mixtures at various concentration ratios, effectively increasing the scope of toxicological assessments beyond what is feasible with in vivo and in vitro methods alone. Thus, the development and refinement of computational models play a key role in advancing our understanding of mixture toxicology and facilitating more effective environmental health risk assessments. These models not only help reduce the reliance on animal testing but also enhance the efficiency and cost-effectiveness of toxicity assessments [4,5].

Quantitative structure–activity relationship (QSAR) methodologies are widely used for assessing the toxicity of chemicals. These methods assess the relationship between chemical structure and their biological activity, providing a toxicological assessment tool. Altenburger et al. have extensively discussed the challenges and methodologies involved in evaluating the toxicity of chemical mixtures and the importance of computational techniques, given the limitations of traditional experimental methods [6]. Luan et al. employed the QSAR approach to model the toxicity of binary mixtures of non-polar narcotic chemicals. They achieved high predictive accuracy, with an R^2^ of 0.94 for their multilinear regression model (MLR) and an R^2^ of 0.96 for the radial basis function neural network (RBFNN) model [7]. This study showed the effectiveness of QSAR models in predicting the toxicological interactions within binary chemical mixtures, indicating their potential application in broader chemical assessment frameworks.

Similarly, Qin et al. developed multiple linear regression models based on different mechanistic assumptions of 24 models using the concentration addition approach and another 24 based on independent action [8]. These models were designed to assess the toxicity of four binary combinations of chemicals across six varying concentration ratios, further showing the applicability of QSAR methodologies in complex mixture toxicity prediction. Toropova et al. also applied QSAR models to predict the toxicity of binary mixtures of benzene and its derivatives, using descriptors calculated from the Simplified Molecular Input Line Entry System (SMILES) [9]. These models tuned to specific chemical categories and achieved high R^2^ values that reflect the adaptability of QSAR in handling diverse chemical datasets and contributing to its limited applicability. However, a critical characteristic of the effective application of QSAR models is defining their applicability domain for the reliability of the predictions [3,10,11,12]. Additionally, the absence of toxicokinetic, toxicodynamic, and pathophysiological mechanisms limits the utility of QSAR models.

Machine-learning (ML) models have emerged recently to predict mixture toxicity. Duan et al., used ink-jet printing (IJP) technology and continuous photographing to generate experimental toxicity data in the form of luminescent inhibition rates (LIRs) [13]. ML-method-based regression models were developed on the ternary mixtures of four compounds at various concentrations to predict the toxicity. The random forest (RF) method gave the best predictive performance, with an average R^2^ of 0.96. The limitation of this strategy was that toxicity prediction could be done only for the mixtures of the compounds in the training set by varying their concentration. Cipullo et al. used neural network (NN) and RF to develop regression models that predicted the toxicity of complex chemical mixtures in two soil samples by first predicting the bioavailability concentration and using the value as input in the toxicity prediction models [14]. This method is applicable for predicting the toxicity for only those two soil samples at a given time *t*. Neither of the models in these two studies can be used generally to predict the toxicity of a mixture of random chemicals. The mixtures comprise a limited number of compounds as the component chemicals, and there is no provision to change the input features based on the descriptor of the component chemicals. These studies apply very specific and complicated methods to generate the toxicity data and use very limited input features to develop ML models with limited applicability. Thus, there is a need for robust models to predict the toxicity of a diverse set of chemical mixtures using easily obtainable descriptors as the input features and a more interpretable form of predicted toxicity. Also, binary classification models that predict whether the mixtures are toxic or not and multiclass classification models that predict the degree of toxicity of the mixtures are important to identify hazardous mixtures.

Moreover, humans are exposed to thousands of potentially dangerous environmental chemicals and mixtures. According to the WHO and the IARC, chemical exposures are responsible for nearly 30% of human cancers. Most diseases, symptoms, or adverse effects are due to chemical mixtures rather than a single chemical, and even less is known regarding the impact of mixture exposure [7,15,16,17,18,19,20,21,22,23,24,25,26,27,28,29,30,31,32,33,34,35,36,37,38,39,40]. Notably, the question of which mixtures contribute to the adverse effects, toxicity, or carcinogenic potential causing the initiation or progression of cancer remains unresolved. Further, for assessing a mixture cancer risk or toxicities, neither computational nor experimental methods currently consider the mechanisms [7,15,16,17,18,19,20,21,22,23,24,25,26,27,28,29,30,31,32,33,34,35,36,37,38,39,40,41,42,43,44]. Therefore, it is important to characterize and understand the characteristic driving markers of toxic and carcinogenic responses of chemical mixtures.

Additionally, the number of possible complex mixture combinations creates significant difficulties for effective biological testing. Consequently, the qualitative and quantitative data assessing the mixtures and their adverse effects are lacking, making translating the existing data into meaningful prevention and therapeutic strategies complex [7,15,16,17,18,19,20,21,22,23,24,25,26,27,28,29,30,31,32,33,34,35,36,37,38,39,40,41,42,43,44]. We recently described an AI–hybrid neural network (AI-HNN) machine-learning method for predicting the binary, multiclass, and categorical carcinogenicity of chemicals and their mixtures in a dose-dependent manner [45,46,47,48]. However, this method does not account for post-exposure effects, such as toxicokinetic and toxicodynamic properties, among other toxicological and pathophysiological characteristics, which limit the utility of this method. We have previously introduced a computational pathophysiology method, termed Chemo-Phenotypic Based Toxicity Measurement (CPTM), which incorporates these properties to predict the toxicity, carcinogenicity, and mechanisms of chemicals and their mixtures [49]. However, the CPTM lacks the ability to consider dose-dependent effects and chemical interactions in mixtures. Consequently, there is an urgent need for a comprehensive alternative new approach methodology (NAM) to thoroughly assess the toxicity, cancer risks, and mechanisms associated with chemical mixtures.

In the present study, our objective is to introduce and validate a novel, comprehensive new approach methodology (NAM) designated as AI-CPTM. This methodology synergistically integrates the Chemo-Phenotypic Based Toxicity Measurement (CPTM) and the hybrid neural network (AI-HNN) models. The AI-CPTM predictions were validated using various performance metrics and validated with data from the literature. Additionally, we conducted validations of the toxicities of chemicals and their mixtures, as well as their chemical interaction effects, using the zebrafish-embryo toxicity assay. In these validations, our AI-HNN and AI-CPTM methodologies demonstrated robust performance in predicting the toxicity of chemicals and their mixtures in a dose-dependent manner.

## 2. Materials and Methods

### 2.1. Collection of Experimental Chemical-Mixtures Data from the Literature

Due to the lack of available animal experimental data on chemical mixtures, we initiated a comprehensive collection of experimental mixture data reported for diverse species. We compiled data for 981 chemical mixtures from various scientific publications. These data were gathered from toxicity studies across multiple species, with all reference citations provided in Supplementary Table S1 all references. The toxicity metrics for these 981 mixtures are presented as either LD50 (median lethal dose), LC50 (median lethal concentration), or EC50 (median effective concentration) values. The data are converted to EC_50_/LC_50_ if given as pEC50/pLC50 and to mg/L if given as mol/L. To convert data from pEC50/pLC50 to EC50/LC50, we used EC_50_ = 10^(−pEC50)^.

Molar mass (or molecular weight in g/mol) is used to convert EC_50_ mixture data from mol/L to mg/L. For a mixture of two chemicals A and B,

*EC*_50_ g/L = *EC*_50_ mol/L (*molar mass A* × *mole fraction A* + *molar mass B* × *mole fraction B*) (1)

Whereas molar masses A and B are the molecular weights of the chemical components A and B in the mixture, mole fractions A and B are the mole fractions of the components A and B in the mixture. The mole fractions of the component chemicals in the mixture were calculated from the median effective concentrations of the component chemicals when acting alone and their corresponding toxicity ratios in the mixture. In some cases, the fractions of the components in the mixture are provided. The detailed computation of mole fractions is described in the regression method section.

### 2.2. Collection of Drug Combinations

Data on drug combinations were downloaded from the Drug Combination Database (DCDB) [50]. Out of 1363 combinations, 942 were binary combinations. The DCC_IDs of each component drug were mapped to DrugBank IDs. Descriptors were calculated for the component drugs of binary combinations.

### 2.3. Collection of ChemIDPlus Single Chemicals

From ChemIDplus, 92,322 single chemicals with LD_50_ values in mg/kg were collected, as detailed in our previous publication [45,47,48]. These chemicals are included with PFAS. We considered rat and mouse oral route of exposure data, resulting in 22,808 single chemicals for which descriptors were calculated.

### 2.4. Creation of Binary-Mixture Dataset

We developed multiple datasets to evaluate the predictive capabilities of machine-learning models in the context of binary chemical-mixture toxicity. The creation of datasets I to VII is guided by distinct objectives, each designed to address specific aspects of binary chemical-mixture analysis. Dataset I establishes a foundational framework by categorizing 981 binary mixtures into toxic and non-toxic groups. Dataset II seeks to balance this initial dataset by including non-toxic binary drug combinations to improve statistical reliability and enhance the training of models by equalizing the distribution of classes. Dataset III further expands the non-toxic data by incorporating additional binary drug combinations to enrich the dataset for comprehensive toxicity characteristic studies. Dataset IV uses a larger chemical library from ChemIDplus to generate a diverse dataset, using different toxic/nontoxic case assumptions that allow more extensive testing of predictive models across a wide range of chemicals. Dataset V integrates virtual mixtures to provide an enriched chemical-mixture training set for the simulation of toxicity predictions. Dataset VI enhances an earlier dataset by adding more mixtures from ChemIDplus, increasing data diversity and improving the robustness of the predictive models. Dataset VII focuses on chemical similarity, selecting compounds based on their similarity to known toxic or non-toxic components. It explores the toxicity of various binary combinations in a large-scale dataset designed for rigorous model testing. Overall, each dataset is designed to enhance the accuracy and applicability of predictive toxicity assessments in chemical mixtures.

Dataset I consists of 981 binary mixtures, consisting of 564 toxic and 382 non-toxic chemical mixtures.

For dataset II, to create a balanced dataset I, we added 200 binary drug combinations to the non-toxic binary-mixture data of dataset I. Thus, we obtained 564 toxic and 582 non-toxic mixtures.

For dataset III, here, all 373 binary drug combinations were added as non-toxic data to the 981 chemical-mixture dataset I. Thus, dataset III consists of 564 toxic and 755 non-toxic binary mixtures.

### 2.5. Generating Virtual Mixtures: Assumptions and Methods

While simulating the dose-dependent toxicity of chemical mixtures, we initially addressed the lack of experimental data on mixtures, including PFAS, by creating virtual mixtures. These mixtures, both binary and multiple, were derived from individual chemicals. As we previously described [45], we employed various permutations and combinations to generate mixture combinations from individual ChemIDplus chemicals, which include emerging PFAS chemicals. Given the impracticality of handling the millions of potential combinations of 22,808 ChemIDplus chemicals, we adopted a representative sampling approach to manage the dataset, as previously outlined [45,48]. This method enables us to capture diverse combinations while effectively reducing the vast number of possibilities. To create virtual mixtures, we employed assumption-based cases to form different combinations of mixtures, as detailed in an earlier publication [45]. In this study, we report results exclusively from Case 1, where virtual mixtures were formed by combining a single toxic chemical with another toxicant to produce a toxic mixture, and Case 2, where mixtures were formed by combining a nontoxic chemical with another non-toxicant to create a nontoxic combination. In this way, we formed the following virtual mixture dataset from the ChemIDPlus chemicals.

For dataset IV, this dataset was based on the 22,682 ChemIDplus chemicals, comprising 6436 toxic and 16,246 non-toxic chemicals. Assumption-based binary mixtures were generated by uniquely combining 6436 toxic chemicals to form 3218 toxic binary combinations, and 16,246 non-toxic chemicals were similarly combined to form 8123 non-toxic binary combinations. Consequently, a total of 11,341 unique binary chemical combinations were created.

For dataset V, comprising 12,293 virtual binary mixtures, this dataset includes 11,341 combinations from ChemIDplus, 373 drug combinations, and 557 additional binary mixtures. Within this set, 3592 are classified as toxic mixtures, while 8701 are considered non-toxic mixtures.

For dataset VI, this dataset size was increased by adding randomly selected 400 toxic and 400 non-toxic ChemIDplus binary combinations to dataset III, ending in 766 toxic and 964 non-toxic mixtures.

For dataset VII, from the pool of 22,808 chemicals in ChemIDplus, 16,320 were identified as non-toxic and 6488 as toxic. Only those chemicals with a Tanimoto similarity >0.6 to the 236 component chemicals from the binary mixtures previously identified in the literature were selected, resulting in 3833 non-toxic and 1659 toxic chemicals. We hypothesized that the binary mixtures of toxic chemicals would invariably be toxic, those comprising only non-toxic chemicals would remain non-toxic, and those combining toxic and non-toxic chemicals would be classified as toxic. From all possible binary combinations, 30,000 were randomly selected as toxic mixtures from the toxic chemical set, 60,000 as non-toxic mixtures from the non-toxic set, and 60,000 as toxic mixtures from the mixed set. This process yielded 90,000 toxic binary mixtures and 60,000 non-toxic binary mixtures. Additionally, 981 experimental binary-mixture data from the literature were incorporated into the 150,000 ChemIDplus mixture dataset.

### 2.6. Hybrid Neural Network (HNN) Method for the Prediction of Chemical-Mixture Toxicity

We used the hybrid neural network (HNN) framework called AI-HNN, which was developed in our previous work to predict dose-dependent single-chemical toxicity and dose-dependent mixture carcinogenicity prediction [47,48]. HNN was developed using the Keras API in Python. A convolutional neural network (CNN) merges with a multilayer perceptron (MLP)-type feed-forward neural network (FFNN) to make the final toxicity prediction of the chemicals. CNN uses a 3D array of one-hot encoded SMILES strings as the input, while the FFNN uses molecular descriptors of the chemicals calculated using QikProp [51] and Mordred [52] as the input. In addition to an input and an output layer, a CNN consists of convolutional, activation, pooling, and fully connected layers. CNN eliminates the requirement of a very high number of neurons and parameters for the input of large size by allowing the network to be deeper but with few parameters. It uses a pooling layer to reduce the data size and helps control overfitting. The final classification is done by implementing the sigmoid activation function in the case of binary classification, the softmax activation function in the case of multiclass classification, and the linear activation function in the case of the regression model.

#### 2.6.1. Dose-Dependent Relationship of the Chemical Mixtures Using the HNN

Next, considerations of the dose-dependent ratio of chemical components in a mixture were included in two steps. In the first step, we modified the concentration addition CA model; in the second step, we used a mathematical approach. The modified CA model involves calculating and integrating dose-dependent ratios for the different chemical components in a mixture. For most cases, mainly for virtual mixtures, the experimental dose-concentration data of a chemical is not available. Therefore, we developed regression models to calculate the concentrations.

##### Calculation of Dose-Dependent Ratio of Chemicals in a Mixture and Computation of Chemical Interaction Effects

Regression models are developed to calculate the predicted median effective concentration (EC_50_) range of the component chemicals in the mixtures. Similarly, LC_50,_ and LC_50_ concentrations were calculated. The regression models were derived by modifying the concentration addition (CA) model [53] for the mixtures described below.

According to the concentration addition (CA) model [53], mixture toxicity is given by

(2) $${EC}_{50mix}=\frac{C_{M}}{\frac{C_{A}}{{EC}_{50A}}+\frac{C_{B}}{{EC}_{50B}}}\;=\;\frac{C_{M}}{TU}$$

where the EC_50mix_ is the median effective concentration of the mixture, and *C_A_*, *C_B_*, and *C_M_* are the concentrations of components A and B and the mixture M that was required to cause the median effect (50% effect). EC_50A,_ and EC_50B,_ are the median effective concentrations of component chemicals A and B when acting as a single compound.

The sum of toxic units (TUs) of each component gives the joint toxicity of the mixture.

(3) $$TU=\frac{C_{A}}{{EC}_{50A}}+\frac{C_{B}}{{EC}_{50B}}\;=\;1$$

From Equations (2) and (3),

(4) $$C_{M}=TU\times {EC}_{50mix}$$

where TU ranging from 0.8 to 1.2 indicates additive effect; TU > 1.2 indicate synergistic effect; TU ≤ 0.8 indicate antagonistic effect; and TU ≥ 0.8 indicate independent action effect.

Here, we systematically modeled the chemical interaction effects, encompassing additive, synergistic, antagonistic, and independent action effects by integrating appropriate toxic unit (TU) ratios. This methodological framework permitted the incorporation of varying doses of the component chemicals and their respective interaction effects, enabling dose-dependent effects. For the scope of this study, we specifically focused and reported results only on the additive effects of the component chemicals within the mixtures while calculating the predicted range of concentrations for the components in the mixture at median inhibition. Thus, when TU = 0.8 to 1.2, then, Equation (3) becomes,

(5) $$C_{M}\;=\;(0.8\times {EC}_{50mix}\;\mathrm{to}\;(1.2\times {EC}_{50mix})$$

Equation (2) can be rewritten as:

(6) $${EC}_{50mix}=\frac{1}{\frac{p_{A}}{{EC}_{50A}}+\frac{p_{B}}{{EC}_{50B}}}$$

where $p_{A}=\frac{C_{A}}{C_{M}}$ and $p_{B}=\frac{C_{B}}{C_{M}}$ are the mass fractions of components A and B in the mixture at median inhibition.

The concentration of components A and B that causes a median effect in the mixture can be calculated in terms of mass fraction as

(7) $$C_{A}=p_{A}\times C_{M}\;C_{B}=p_{B}\times C_{M}$$

Thus, from Equations (5) and (7), we get the final Equation (8) to calculate the range of concentration of each component chemical required to cause the median effect by the mixture as:

(8) $$C_{A}\;=(p_{A}\times 0.8\times {EC}_{50mix})\;\mathrm{to}\;(1.2\times {EC}_{50mix}\times p_{A})\;C_{B}\;=(p_{B}\times 0.8\times {EC}_{50mix})\;\mathrm{to}\;(1.2\times {EC}_{50mix}\times p_{B})$$

#### 2.6.2. Dose-Dependent Computations Using Mathematical Approach

The dose consideration during mixture formation uses the concentration information of each component chemical that makes the mixture calculate the mixture descriptor. We used the reported concentration information associated with a chemical while making mixture combinations. In cases where concentration information was unavailable for certain chemicals, such as some experimented chemicals and virtual mixture chemicals, we assigned concentrations that we computed from the modified concentration addition model, or we assigned equal concentrations. In this study, we report results for the equal concentrations assigned for the component chemicals. We also report only the binary-mixture data.

As described in Section 1 on data collection, although datasets I to VII are prepared by combining data from various sources, the toxicity determination metrics and thresholds vary across and within these datasets. The 981 experimental mixtures’ data collected from the literature consist of LC_50_ and EC_50_ data. We used one standard cutoff of 100 mg/L for a toxicity determination set by EPA. In the case of the LD_50_ data of ChemIDPlus chemicals, we used a cutoff of 500 mg/kg for determining the toxicity. All the collected chemical experimental data were converted to mol/L before calculating the log (1/EC_50_, LC_50_, or ED_50_).

Next, we considered dose dependency by using mole fractions and molecular descriptors of chemicals, which are given as input features for the HNN FFNN framework. These features enabled us to calculate the dose-dependent factor, ‘D’. Mole fractions of the component chemicals in a mixture were calculated from their median effective concentration when acting alone and their corresponding toxicity ratio in the mixture. The dose-dependent chemical-mixture descriptor ‘*D*’ was calculated using three different mathematical methods formulas (sum, difference, and norm) as the basis, as described previously [45,54].

For the *sum*, the mixture descriptor dose-dependent factor ‘*D*’ is calculated as the sum of the molecular descriptors $d_{1}$, $d_{2}$…$d_{n}$ of the two or more component chemicals in a mixture weighted by their respective mole fractions $x_{1}$, $x_{2}$…$x_{n}$ in a mixture.

*D* = *x*_1_*d*_1_ + *x*_2_*d*_2_ + ⋯⋯ *x*_n_*d*_n_ (9)

In this study, we used and reported only the sum method results. The mixture descriptor dose-dependent factor ‘D’ was calculated using the sum method. We used 653 descriptors for each component chemical, computed using the Mordred [52]. The mixture descriptor ‘*D*’ was calculated as the sum of the molecular descriptors ‘*d*1’ and ‘*d*2’ of the two-component chemicals of the mixture, each scaled by their respective concentration fractions ‘*x*1’ and ‘*x*2’ in mg/L.

### 2.7. Molecular Structural Feature Descriptors Using SMILES of the Chemicals

Next, the SMILES structural representation and image bytes of the chemicals are computed, which are used as input features for the HNN CNN model. For the SMILES, the mixture SMILES ‘*S*’ is generated by the concatenation of the two SMILES strings ‘*S*1’ and ‘*S*2’ with a period (.) as the separator.

(10) S=S1.S2

### 2.8. SMILES Preprocessing

The detailed process is explained in our previous studies for single-chemical toxicity and mixture carcinogenicity studies [45,47,48]. However, here, we used a slightly modified process. Molecular SMILES are used for chemical nomenclature using ASCII strings to represent the 2D structural attributes that we used here as the input for our CNN models. Since raw texts cannot be directly used as input for the deep-learning models, we encoded them as numbers. The entire list of SMILES strings is first represented on the tokenizer to create a dictionary of the set of all the possible characters in the SMILES string and their corresponding index. We assumed and created a dictionary ‘D’, where

D = {‘C’: 1, ‘=‘: 2, ‘(‘: 3, ‘)’: 4, ‘#’: 5, ‘N’: 6, …, ‘ ‘: M } (11)

This results in every character in the SMILES string being assigned a unique integer value, which is the index of the character in the dictionary. The SMILES entry for every chemical is then converted to a one-hot encoded 2D matrix. For example, an acrylonitrile-d3 chemical with SMILES string C=CC#N is one-hot encoded as:

(12) $$\left[\begin{array}{c} C\\ \begin{array}{c} C\\ C\\ \begin{array}{c} N \end{array} \end{array} \end{array}\right]\;=\;\left[\begin{array}{ccc} \begin{array}{ccc} \begin{array}{cc} 1 & 0 \end{array} & \begin{array}{cc} 0 & 0 \end{array} & \begin{array}{cc} 0 & 0 \end{array}\\ \begin{array}{cc} 0 & 1 \end{array} & \begin{array}{cc} 0 & 0 \end{array} & \begin{array}{cc} 0 & 0 \end{array}\\ \begin{array}{c} \begin{array}{cc} 1 & 0 \end{array}\\ \begin{array}{cc} 1 & 0 \end{array}\\ \begin{array}{c} \begin{array}{cc} 0 & 0 \end{array}\\ \begin{array}{cc} 0 & 0 \end{array} \end{array} \end{array} & \begin{array}{c} \begin{array}{cc} 0 & 0 \end{array}\\ \begin{array}{cc} 0 & 0 \end{array}\\ \begin{array}{c} \begin{array}{cc} 0 & 0 \end{array}\\ \begin{array}{cc} 0 & 0 \end{array} \end{array} \end{array} & \begin{array}{c} \begin{array}{cc} 0 & 0 \end{array}\\ \begin{array}{cc} 0 & 0 \end{array}\\ \begin{array}{c} \begin{array}{cc} 1 & 0 \end{array}\\ \begin{array}{cc} 0 & 1 \end{array} \end{array} \end{array} \end{array} & \cdots & \begin{array}{c} 0\\ 0\\ \begin{array}{c} 0\\ 0\\ \begin{array}{c} 0\\ 0 \end{array} \end{array} \end{array}\\ \vdots & \ddots & \vdots \\ \begin{array}{ccc} \begin{array}{cc} 0 & 0 \end{array} & \begin{array}{cc} 0 & 0 \end{array} & \begin{array}{cc} 0 & 0 \end{array} \end{array} & \cdots & 0 \end{array}\right]$$

A 3D matrix of size K × L × M is obtained eventually, where K is the number of chemicals, L is the maximum length of the SMILES string, and M is the number of sets of all the possible characters in the SMILES string in the K chemicals. One-hot encoding means converting the integer value of each character in the SMILES to its equivalent binary vector of length M.

### 2.9. Descriptor Calculation

We computed 653 descriptors for each component chemical using the Mordred 2022 software [52]. Additional descriptors were computed from the SMILES of the component chemicals. The structconvert utility in Schrodinger 2023 software [51] was used to convert the SMILES of the chemicals to 2D structures in .sdf format. The 2D .sdf file was converted to 3D structures using a 3D minimization application in Schrodinger’s 2023 Canvas software. Additional descriptors based on a 3D molecular structure, such as ADME (absorption, distribution, metabolism, and excretion) properties, such as the octanol–water partition coefficient, MDCK cell permeability, Caco-2 cell permeability, binding to human serum albumin, and human oral absorption, were calculated using QikProp application in Schrodinger 2023 software [51]. In the last step, these descriptors were given as input features for the FFNN and CNN of the HNN hybrid framework, and simulations were initiated. The output of the CNN and FFNN are merged within the HNN framework [45,46,47,48] to create mixture classification models, which are described below. Eventually, we predicted the unknown chemical-mixture toxicity in a dose-dependent manner with the inclusion of the chemical interaction effect.

### 2.10. Binary Classification Criteria

According to the EPA’s toxicity categories, a concentration of less than 100 mg/L is considered toxic [55]. Therefore, all chemicals with EC_50_/LC_50_ values greater than or equal to 100 mg/L were considered non-toxic. For binary classification, out of 981 binary mixtures, 610 were classified as toxic, while 371 were classified as non-toxic.

### 2.11. Multiclass Classification Criteria

Multiclass models predict the degree of toxicity of a binary mixture of chemicals by classifying each mixture into one of the five classes. The EPA classifies pesticides into five categories based on the degree of toxicity [55]. We applied these EPA classification criteria to the EC_50_, LC_50_, and LD_50_ concentrations for various organisms. For example, the acute toxicity classification for aquatic organisms is based on concentrations in mg/L, categorizing compounds as:

- very highly toxic (<0.1 mg/L);
- highly toxic (0.1–1 mg/L);
- moderately toxic (>1–10 mg/L);
- slightly toxic (>10–100 mg/L);
- practically nontoxic (>100 mg/L).

Using these categorization criteria from the EPA for different concentrations and organisms, we classified 981 experimental binary mixtures accordingly, as follows: 371 are very highly toxic (class 4), 273 are highly toxic (class 3), 153 are moderately toxic (class 2), 70 are slightly toxic (class 1), and 114 are practically non-toxic (class 0). Similarly, we classified the mixture datasets I to VII (see Section 4 and Section 5).

### 2.12. Developing Binary and Multiclass Classification Models Using Other Machine-Learning Methods

To compare and evaluate the binary and multiclass predictions of our HNN method, we developed binary and multiclass classification models using various machine-learning techniques, including random forest (RF), bagged decision trees (also known as bootstrap aggregating or bagging), and adaptive boosting (AdaBoost). These models were then combined to create an ensemble model for improved predictive performance.

### 2.13. Developing Regression Models Using Other Machine-Learning Methods

To compare and evaluate the regression-based potency predictions of the HNN, we developed regression models using various machine-learning methods, including random forest (RF), support vector regressor (SVR), gradient boosting (GB), kernel ridge (KR), decision tree with AdaBoost (DT), and KNeighbors (KN). These models were implemented using the scikit-learn package in Python to generate the final consensus prediction of the median effective concentration (ED_50_ or EC_50_). A consensus value is calculated based on the average predicted values of all seven models.

### 2.14. Ensemble Model

The ensemble of model predictions optimizes the predictive performance of models and was employed in the binary classification models. The ensemble prediction, as we described previously [45,47,48], was used to calculate the final prediction based on the prediction results from HNN, RF, bagging, and AdaBoost.

### 2.15. Robust Model Evaluation

#### Binary and Multiclass Classification Model Evaluation

The results presented here are an average of 30 iterations. Approximately 20% of the data were randomly separated from the datasets as the test set in each iteration. We employed a robust evaluation process to assess the performance of the mixture classification models. Initially, about 20% of the available data were randomly allocated as the test set for each iteration to ensure an unbiased assessment. This evaluation process was repeated for 30 iterations, and the average results were used to evaluate the model’s performance. Several metrics were used to assess the classification models. Stratified 10-fold cross-validation (CV) was performed for the classification models, and the average of 10 CV results was calculated. Stratified cross-validation ensures that the proportion of samples for each class is maintained while selecting the test set. The performance of each model was evaluated based on accuracy, AUC, sensitivity, and specificity, as we previously described. Accuracy, which measures the proportion of correctly classified instances, served as the primary evaluation metric (Supplementary Equation (S1)). Additionally, the performance of each model was evaluated based on the AUC. AUC provides the probability of a positive outcome being ranked before a negative outcome and is a superior metric for assessing binary classifiers compared to accuracy. Sensitivity, representing the true-positive rate, and specificity, representing the true-negative rate, were also considered to assess the model performance. These metrics offered us insights into the model’s ability to correctly identify positive and negative outcomes within the dataset. Micro-averaging is used in multiclass classification to calculate the average value across all classes by converting the data into multiple binary classes and assigning equal weight to each observation. This technique involves converting the data into binary classes and giving equal weight to each observation, enabling a fair evaluation of the model performance across multiple classes. By considering the average performance across all classes, we gain a comprehensive understanding of the model’s overall classification accuracy and performance.

#### Regression Model Evaluation

Approximately 20% of the data were randomly separated as the test set. The calculated performance metrics of the models were based on an average of 30 iterations. Mean square error (MSE), mean absolute error (MAE), and coefficient of determination (R^2^) were the metrics used to evaluate the performance of the models (Supplementary Equation (S2)).

#### Compound Out

The “compound out” (CO) method for segregating the test set has also been adopted as a means to validate our models with increased rigor. This approach ensures that at least one of the component chemicals within each mixture of the test set is not included in the training set. This methodological choice enhances the robustness of our validation process by testing the model’s predictive ability on entirely new or novel chemicals, thereby mitigating the risk of overfitting and ensuring that the model’s predictions are generalizable to new, unseen compounds. The inclusion of a varied set of chemical and drug mixtures enhances the test set’s complexity and challenges the model’s ability to generalize across a diverse chemical interaction. By evaluating the model performance on this expanded and varied test set, we assess its robustness and accuracy in predicting the toxicity of diverse chemical combinations under different contexts, confirming its applicability and reliability in practical, real-life scenarios. By implementing this stringent validation technique, we demonstrate the model’s capacity to accurately predict chemical interactions and mixture toxicities.

For *COSet* I, all 61 binary mixtures comprising four specific chemicals, such as sulfamonomethoxine, sulfachloropyridazine, trimethoprim, and 2,4-dichlorophenol, were included in the test set of dataset II. Additionally, 19 binary combinations of approved drugs were integrated to further diversify the test set, obtaining a total of 80 distinct mixtures designated for the test set.

For *COSet* II, all 60 binary mixtures, consisting of seven specific chemicals such as Penicillin V potassium salt, benzene, gamma valerolactone, sulfapyridine, sertraline, p-dinitrobenzene, and diazinon, were included in the dataset II test set. To enhance the diversity and complexity of the test set, an additional 20 binary drug combinations were incorporated, bringing the total to 80 distinct mixtures.

### 2.16. Reproducibility

The rigor and reproducibility were discussed in the above sections. The model and the outputs are reproducible with our collected data. Also, the simulations begin with a fixed seed for reproducibility.

### 2.17. AI-CPTM Score Computations

The AI-CPTM score is computed as the combination of the AI-HNN and CPTM scores.

AI-CPTM score = AI-HNN score (binary class + categorical class + potency) + CPTM Z score.

In the case of the AI-HNN score, the binary score is assigned by:

Binary score = binary value 0 or 1: score 1 if carcinogenic or toxic and score 0 if non carcinogen or nontoxic.

The categorical scores are assigned according to the IARC classification for the range of values <1 mg/kg to >2000 mg/kg or <1 mg/L to >500 mg/L, as below:

Categorical score = score 1 for group 1 (carcinogenic or high toxic);

score 0.75 for group 2A (probable carcinogen or toxic);

score 0.5 for group 2B (possible carcinogen or medium toxic;

score 0.25—group 3 (may be a carcinogen or low toxic);

score 0—group 4 (noncarcinogen or nontoxic).

The potency scores are assigned for the range of values <1 mg/kg to >2000 mg/kg or <1 mg/L to >500 mg/L, as below:

Potency score = score 1 for <1 mg/kg/day to <100 mg/kg/day;

score 0.75 for >100 mg/kg/day to <250 mg/kg/day;

score 0.5 for >250 mg/kg/day to <500 mg/kg/day;

score 0.25 for >500 mg/kg/day to <1500 mg/kg/day;

score 0 for >1500 mg/kg/day.

Basically, the total AI-CPTM score cannot exceed a value of 4 because each score is normalized to 1, with four score components in the total score, as seen in the equation below. For example, in the case of highly toxic or carcinogenic mixtures, a typical AI-CPTM total score is given by:

AI-CPTM score = score [(AI-HNN + CPTM)] = score [(1 + 1 + 1) + (>0.9)] = >3.9

## 3. Results and Discussion

The results are presented in two sections. Section I discusses the evaluation of dose-dependent toxicity of chemical mixtures using our hybrid neural network (HNN) method, including comparisons with other machine-learning methods. Section II describes how the HNN is integrated with the CPTM and the integrated AI-CPTM assessment of the dose-dependent toxicity of chemical mixtures. Section III describes the validations of the AI-CPTM predictions.

I.1. Dose-Dependent Toxicity Assessment of Chemical Mixtures Using HNN and other Machine-Learning Methods

*Descriptor calculation for the virtual mixtures using sum, diff., and norm methods.*

We evaluated the dose-dependent toxicity of chemical mixtures using a combination of the existing experimental data, drug combinations, and virtual mixtures generated to supplement the lack of experimental data. To predict binary and categorical toxicity, we developed binary and multiclass classification models using data from 981 experimental binary mixtures sourced from 60 articles (see Supplementary Material S1). Additionally, regression models were developed by modifying the concentration addition model to determine the toxic potency (pEC_50_/pLC_50_) of these mixtures. Of the total data, 785 data (80%) were designated as the training set, with the remaining 196 data set aside for validation. We also explored the impact of sum, difference, and normalization methods on toxicity evaluation, integrating both experimental and virtual data, which yielded highly accurate results. Statistical analyses, including *t*-tests, revealed no significant differences in model performance across accuracy, sensitivity, specificity, precision, and AUC for these methods. All the simulations were carried out over 30 iterations, with stratified 10-fold cross-validation to ensure statistical robustness. Here, we report the results obtained from the sum method.

I.2. Machine-Learning Model Performance using Literature-Derived Experimental Mixtures Data

*I.*2.1*. Mixture Toxicity Prediction using Binary Classification*

**Figure 1:**
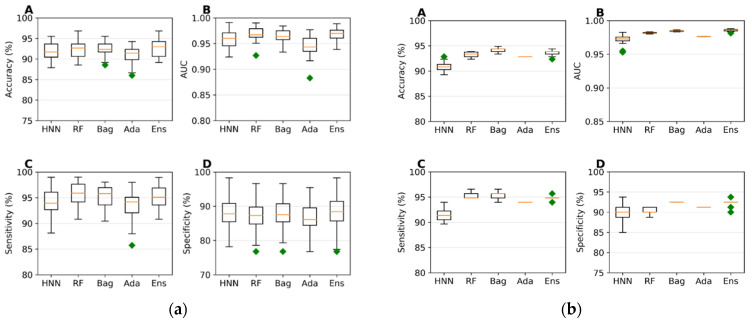
(**a**). (A) Accuracy, (B) AUC, (C) sensitivity, and (D) specificity for the data in the training set achieved by the binary classification models based on HNN, RF, bagging, AdaBoost, and ensemble methods. (**b**). (A) Accuracy, (B) AUC, (C) sensitivity, and (D) specificity for the validation set achieved by the binary classification models based on HNN, RF, bagging, AdaBoost, and ensemble methods. Green symbols indicate far most outlier.

The predictive capability of the machine-learning models was evaluated using a training set of 785 data (494 toxic and 291 non-toxic). A random selection of 157 samples (20%) served as the test set, while the models were trained on the remaining 628 samples during each simulation. The HNN, RF, bagging, AdaBoost, and ensemble were used. The average accuracies achieved ranged from 90.91% to 92.48%, and the average area under the curve (AUC) scores varied from 0.94 to 0.96 across the different models, with the ensemble method exhibiting the highest sensitivity and specificity (Figure 1a). These models were further validated against an external validation dataset of 196 data (116 toxic and 80 non-toxic), where the accuracies ranged from 90.85% to 94.23%, and the AUC scores varied between 0.972 and 0.985. The ensemble method again demonstrated superior sensitivity and specificity, confirming its robustness (Figure 1b).

*I.*2.2*. Mixture Toxicity Prediction using Multiclass Classification*

**Figure 2:**
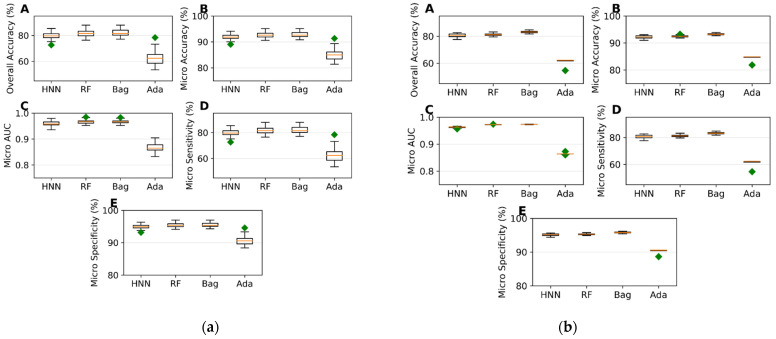
(**a**). (A) Overall accuracy, (B) micro accuracy, (C) micro AUC, (D) micro sensitivity, and (E) micro specificity for the data in the training set achieved by the multiclass classification models based on HNN, RF, Bagging, and AdaBoost methods. (**b**). (A) Overall accuracy, (B) micro accuracy, (C) micro AUC, (D) micro sensitivity, and (E) micro specificity for the validation set achieved by the multiclass classification models based on HNN, RF, Bagging, AdaBoost and Ensemble methods. Green symbols indicate far most outlier.

To evaluate multiclass classification models, we randomly selected 157 samples (20%) from a total of 785 data points (comprising 291 of class 0, 225 of class 1, 122 of class 2, 55 of class 3, and 92 of class 4) as the test set, using the remaining 628 samples as training data. The HNN, RF, bagging, and AdaBoost demonstrated robust performance metrics, with no method falling below significant predictive accuracy (ranging from 62% to 82%), AUC values (86% to 97%), micro-sensitivity (62% to 82%), and micro-specificity (90% to 95%) (Figure 2a). These models were then validated against an external dataset of 196 samples (80 class 0, 48 class 1, 31 class 2, 15 class 3, and 22 class 4) using the same total data pool of 785. The predictive performance continued to show similar accuracy, AUC, micro-sensitivity, and micro-specificity (Figure 2b).

*I.*2.3*. Mixture Toxicity Prediction using Regression Models*

**Figure 3:**
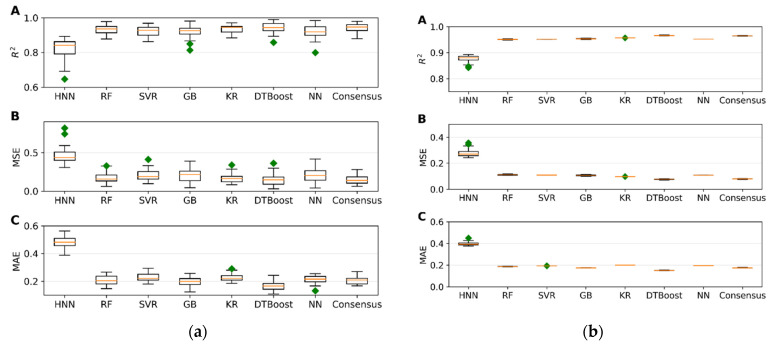
(**a**). (A) Coefficient of determination (R^2^), (B) mean squared error (MSE), and (C) mean absolute error (MAE) for the data in the training set achieved by the regression models based on HNN, RF, SVR, GB, KR, DTBoost, NN and consensus methods. (**b**). (A) Coefficient of determination (R^2^), (B) mean squared error (MSE), and (C) mean absolute error (MAE) for the validation set achieved by the regression models based on HNN, RF, SVR, GB, KR, DTBoost, NN, and consensus methods.

The regression models were evaluated using 645 mixtures, with 129 randomly selected as a test set and the remaining 516 data used for model development. The models were built using HNN, RF, support vector regression (SVR), gradient boosting (GB), kernel ridge (KR), decision tree boosting (DTBoost), and neural network (NN) to determine and compare the toxic potencies. All methods demonstrated robust regression performance metrics, including R^2^, mean squared error (MSE), and mean absolute error (MAE) values, which are indicative of mixture toxic potency prediction accuracy (Figure 3a). Upon testing these models against an external validation set of 160 data, consistently high R^2^ values and low error rates were observed, confirming the model’s robust predictive ability (Figure 3b). Additionally, the predicted range of the concentrations of the component chemicals required to achieve a median effect was calculated from the toxicity values obtained by the HNN and the consensus method (Supplementary Table S2).

*I.*2.4. *Comparison of Mixture toxicity Prediction with the Existing Literature*

Although there is a lack of directly comparable data, our models exhibit broader applicability and enhanced predictive power relative to the existing models, such as those developed by Duan et al. [13] and Cipullo et al. [14], which are confined to a limited number of specific compounds and conditions. Duan et al. formulated regression models to predict toxicity, expressed as luminescent inhibition rates (LIRs), for mixtures of four compounds, relying solely on the concentrations of the constituent chemicals as the input features. Cipullo et al. developed regression models for two distinct soil samples, incorporating soil type, amendment type, chemical concentrations, and time ‘*t*’ as the input features. In contrast, our approach utilizes a more extensive variety of chemical mixtures and employs a broader set of input features, thus enhancing predictive accuracy across a diverse range of toxicological outcomes.

*I.*2.5a*. Evaluation of Machine-Learning Model Performance using Data Derived from Combinations of Experimental Mixtures and Drug-Combination Datasets (Datasets I—III)*

**Figure 4:**
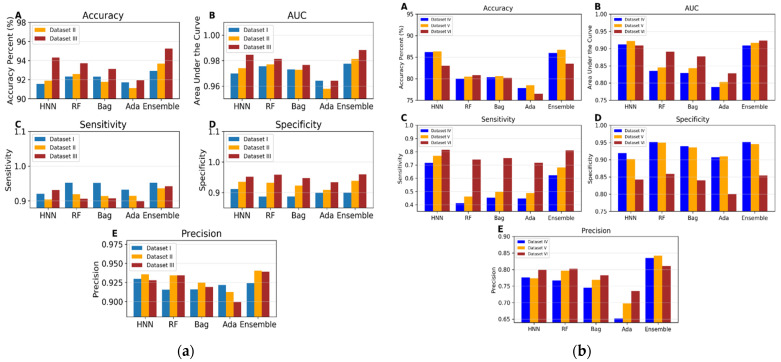
(**a**): Prediction accuracy, sensitivity, specificity, precision, and AUC for the mixture dataset I (no drug combination) with 175 random mixtures in the test set, II (with 100 drug combinations) with 200 random mixtures in the test set, and III (with all 373 drugs combinations) with 250 random mixtures in the test set by HNN, RF, bagging, AdaBoost and the ensemble methods. (**b**): Statistical summary of the results of HNN, RF, bagging, AdaBoost, and ensemble methods with prediction accuracy, sensitivity, specificity, precision, and AUC for the mixture datasets IV, V, and VI are shown.

In the three datasets (I, II, and III), the number of toxic chemicals remains constant, with variations arising only from the addition of non-toxic chemicals through drug combinations (see Materials and Methods section). For the HNN, the specificity improved from 0.91 in dataset I to 0.93 and 0.95 in datasets II and III, respectively. Sensitivity initially decreased from 0.92 to 0.91 and then increased to 0.94 (Figure 4a). The increase in specificity was statistically significant, as indicated by the *p*-values from *t*-tests; however, the changes in sensitivity were not statistically significant. For the RF model, the specificity increased from 0.88 to 0.93 and 0.95, while sensitivity decreased from 0.95 to 0.91 and further to 0.90 (Figure 4a). The increase in specificity was highly significant for both the RF and bagging models. For AdaBoost, the increase in specificity from dataset I to II was not statistically significant, but it was significant between datasets II and III. The decreases in sensitivity were significant across all models, except for HNN. The general rise in specificity across the models can be attributed to the inclusion of additional drug data, which are negative samples in the datasets. These data were predicted with nearly 100% accuracy, enhancing the overall true-negative rate. Conversely, the decline in sensitivity for most models was likely due to a decreased prediction capability for positive samples, potentially resulting from overfitting to the training sets comprised predominantly of negative samples. The HNN sensitivity had the most negligible impact on all the models. The HNN results demonstrated no significant difference in sensitivity between datasets I and II or between datasets I and III. The enhancement in HNN prediction accuracy with dataset III compared to dataset I was statistically highly significant, attributed to an increase in specificity and a non-significant decrease in sensitivity. There was a significant increase in the prediction accuracy of RF. Still, the increase in prediction accuracy for bagging and AdaBoost, with the addition of drug combination data was not statistically significant (Figure 4a).

*I.*2.5b*. Toxicity Prediction using Binary Classification with Virtual Mixtures and Drug-Combination Datasets (Datasets IV to VI)*

Dataset IV consists of 3226 toxic and 8137 non-toxic binary combinations derived from the single-chemical data of ChemIDplus. Dataset V includes 3592 toxic and 9258 non-toxic binary combinations, supplemented by 373 binary drug combinations and 557 binary chemical mixtures. The toxicity prediction results from these two datasets were very similar, as shown in Figure 4b. Further, to investigate whether the large number of ChemIDplus chemical combinations influenced the results, toxicity predictions were conducted using dataset VI, which comprises only 800 ChemIDplus chemical combinations, alongside 373 binary drug combinations and 557 binary chemical mixtures. The HNN maintained an accuracy of 86% with both datasets IV and V. For dataset IV, the HNN demonstrated a sensitivity of 0.71, a specificity of 0.91, a precision of 0.77, and an AUC of 0.91. The accuracy of HNN slightly decreased to 83% for dataset VI, but the AUC remained at 0.91. The performance of other machine-learning methods was consistent across all datasets, though their AUCs were notably higher for dataset VI. Further, the results with dataset VI suggest that the observed decrease in accuracy from integrating ChemIDplus combination data to form dataset V from dataset III was not due to the large size of the ChemIDplus data, which also yielded lower accuracy. This reduction in accuracy may be due to the increased diversity of chemicals in the training and test sets for datasets V and VI compared to datasets I, II, and III.

*I.*2.6. *Compound-Out Method*

**Figure 5:**
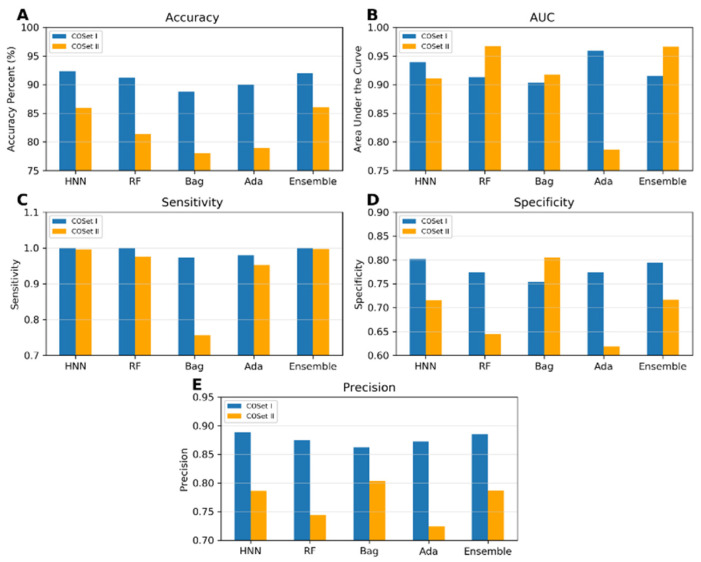
Prediction accuracy, sensitivity, specificity, precision, and AUC for the compound-out mixture COSet I and COSet II by HNN, RF, bagging, AdaBoost, and the ensemble methods.

The models were constructed using the COSet I and II datasets and validated with the more stringent compound-out method. The accuracies achieved were 92.33%, 91.2%, 88.79%, 90%, and 92.04% for the HNN, RF, bagging, AdaBoost, and ensemble methods, respectively, for COSet I and 85.92%, 81.42%, 78%, 79%, and 86%, respectively, for COSet II (Figure 5). The sensitivity of the models approached a value very close to one across all methods, except for the bagging method for COSet II. The average specificities were 0.81, 0.77, 0.75, 0.77, and 0.79 for COSet I and 0.72, 0.64, 0.80, 0.62, and 0.71 for COSet II, respectively. These results demonstrate the model’s robust predictive capabilities, even for new chemicals. The HNN model exhibited superior accuracy, AUC, sensitivity, and precision for both COSet I and II, whereas the bagging method performed well in predicting specificity and precision (Figure 5).

**II.**  **AI-CPTM: the integration of the HNN Machine-Learning Method with the CPTM Pathophysiology Method for the Assessment of Dose-Dependent Toxicity of Chemical Mixtures**

We employed the HNN and CPTM, as well as the integrated AI-CPTM approach, for toxicity predictions concerning single chemicals and mixtures. We previously introduced the CPTM pathophysiology method for predicting the toxicity and carcinogenicity of hazardous chemicals [49]. The CPTM, a proteo-chemometric pathophysiology method, predicts phenotypic responses and model interactions between chemicals (and their mixtures) with genes and cells within physiological processes. It also identifies the chemicals that are predicted to interact with the key cellular networks associated with toxicity or cancer, by estimating the risks in terms of a toxic or cancer risk Z score. Additionally, our earlier work introduced an HNN machine-learning-based framework to predict mixture toxicity and carcinogenesis that demonstrated a higher prediction accuracy. However, we noted a significant reduction in the predictive accuracy of our HNN approach for carcinogenic mixtures when transitioning from a random to a distinct separation of training and test datasets [45,46,47,48]. This decline was due to the absence of biological-specific variables such as toxicokinetics (TK), toxicodynamics (TD), mechanisms, and the complex behavior of chemical mixtures. Further, relying exclusively on HNN to predict specific organ toxicity or cancer types is inadequate. We developed an integrated approach that incorporates these factors to address these limitations, targeting toxicology and carcinogenesis endpoints. The combined HNN and CPTM, termed the AI-CPTM method, integrates the toxicity score from HNN with the CPTM Z score. This combination identifies potentially toxic or carcinogenic chemicals or mixtures, elucidates potential mechanisms, and determines specific organ toxicity or carcinogenicity. The HNN method assigns a binary toxicity status (zero for non-toxic, one for toxic), while the CPTM outputs a Z score, where higher scores indicate greater toxicity. Detailed methodologies for Z score computation by CPTM and descriptor calculation, along with toxicity and carcinogenicity predictions by HNN for single chemicals and mixtures, have been reported in our published studies [49]. This paper exclusively presents the outcomes of toxicity predictions made using the CPTM, HNN, and the combined AI-CPTM methods for single chemicals and binary mixtures, which are discussed below.

*II.1.*  *Single-chemical Toxicity—Binary Classification*

We initiated our evaluation by assessing the performance of the AI-CPTM method using 21,758 rat and mouse oral LD_50_ data obtained from ChemIDPlus as a training dataset. A unique set of 1050 chemicals served as the test set, for which molecular descriptors were calculated. The lowest effective level (LEL) of chemical dose was considered to determine toxicity. We employed various LD_50_ thresholds, such as 50 mg/kg, 250 mg/kg, 500 mg/kg, 750 mg/kg, and 1500 mg/kg, as previously described [45,47,48]. Toxicity predictions were made for the experimentally known 1050 toxic chemicals using the HNN and CPTM methods.

II.1.1. Accuracy based on Experimental Toxicity

To determine whether combining HNN machine-learning predictions with CPTM predictions enhances the performance of toxicity predictions, we performed an experimental comparative analysis. This analysis involved individual CPTM predictions and the combined CPTM + HNN predictions, i.e., AI-CPTM, using the experimentally known toxic set of 1050 chemicals. We counted the experimentally determined toxic chemicals against the CPTM Z-score-ranked toxic chemicals, both with and without the inclusion of HNN predictions, and calculated the percentage of correctly predicted toxic chemicals.

*CPTM performance without HNN predictions added*

**Figure 6:**
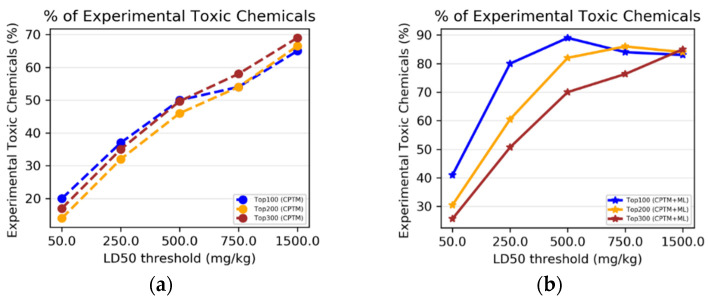
The percentage of experimentally determined toxic chemicals counted (**a**) against CPTM’s Z-score ranking of chemicals, (**b**) counted against CPTM’s Z-score ranking obtained after adding ML (AI-HNN) score (AI-CPTM).

Chemicals were sorted in descending order from higher (more toxic) to lower (less toxic) values based on the CPTM Z score. The top-ranked 100, 200, and 300 chemicals were marked. This implies that all top 300 chemicals are relatively toxic, with the top 1 being the most toxic. We, then, annotated the experimentally assessed toxic or non-toxic outcomes for the top 300 chemicals (toxic = 1, non-toxic = 0). To determine the number of correctly predicted chemicals by the CPTM in the top 100, 200, and 300, we counted the number of ‘1’s known to be toxic from experimental studies. The results, displayed in Figure 6a, show the percentage of experimentally determined toxic chemicals against the CPTM’s Z-score ranking. Figure 6a demonstrates that the percentage of chemicals correctly identified as toxic at various Z-score cutoffs (e.g., top 100, 200, 300) did not vary significantly. The trend of correctly predicting the experimental results increased with increasing the LD_50_ thresholds, with the highest prediction accuracy of 69% achieved at the 1500 mg/kg threshold. This trend suggests that the CPTM is more accurate at identifying toxic chemicals among those with higher Z scores. On the other hand, a decreasing and inconsistent trend across different toxicity thresholds could indicate the model’s predictive limitations. These findings establish the baseline effectiveness of the CPTM for assessing chemical toxicity without the enhancements from machine-learning HNN. The strategy of ranking chemicals by their Z scores and then correlating them with experimental outcomes offers a direct method to evaluate the model’s predictive accuracy. Using only the CPTM Z score for this analysis provides a benchmark for comparing the performance of the AI-enhanced CPTM, which incorporates HNN predictions, as detailed below.

*CPTM performance with HNN added (AI-CPTM)*

The study was expanded by integrating HNN machine-learning predictions into the CPTM. As detailed in the AI-CPTM score computations section, the new total score was calculated by adding a value of 1 to the CPTM Z score for chemicals predicted to be toxic by the HNN method. The effectiveness of this AI-enhanced CPTM (AI-CPTM) was assessed by counting the number of correctly predicted toxic chemicals among the top 100, 200, and 300 and, then, annotating the experimentally assessed toxic or non-toxic outcomes for the top 300 chemicals, and the findings are displayed in Figure 6b. Figure 6b shows the percentage of experimentally determined toxic chemicals, as per the AI-CPTM (CPTM+ML) Z-score ranking. That is, the percentage of chemicals correctly identified as toxic at different Z-score thresholds (e.g., top 100, 200, 300) after integrating HNN predictions into the CPTM. The AI-enhanced CPTM’s performance in predicting toxic chemicals did not differ significantly for the top 100, 200, and 300 ranked chemicals. Moreover, the accuracy of predicting experimental results improved with higher LD_50_ thresholds, with the highest accuracy reached at the 500 mg/kg threshold. For the 1500 mg/kg threshold, the AI-CPTM showed a similar trend in prediction accuracy for its top 100, 200, and 300 ranked compounds, as observed in the CPTM alone. An increasing overall prediction performance trend suggests that the CPTM more accurately identifies toxic chemicals at a higher Z score.

Conversely, a decreasing and inconsistent trend across different toxicity thresholds could indicate the model’s predictive limitations. The AI-CPTM minimum prediction accuracy (Figure 6b) starts at 41% for the 50 mg/kg threshold and reaches up to 89% at 500 mg/kg, compared to 20% and 69%, respectively, for the traditional CPTM (Figure 6a). These findings indicate that incorporating HNN predictions significantly enhances the CPTM’s ability to predict chemical toxicity. By using machine learning, the AI-CPTM is expected to provide more precise and refined toxicity predictions, potentially revealing subtle patterns and correlations not detectable with the conventional CPTM or standalone HNN.

II.1.2. Accuracy Based on HNN Predicted Toxicity

Next, we sought to determine whether integrating predictions from the HNN with those from the CPTM enhances the overall accuracy of toxicity predictions. We performed a comparative analysis of individual CPTM predictions and the combined CPTM + HNN predictions (AI-CPTM) and evaluated the stand-alone HNN predictions for 1050 chemicals. We calculated the percentage of toxic chemicals predicted by the HNN. We compared it against the chemicals ranked by the CPTM Z score, both with and without including the HNN predictions.

*CPTM Performance without HNN Added*

**Figure 7:**
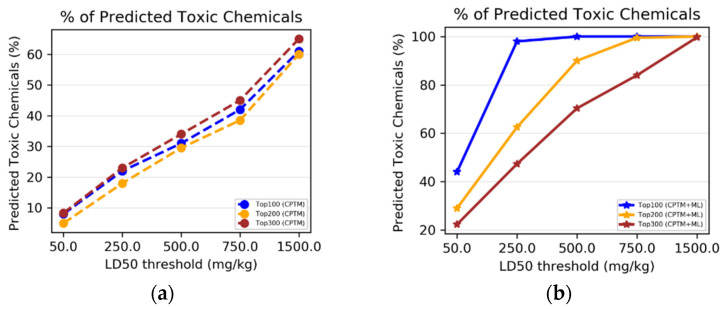
(**a**) Percentage of machine-learning-based predicted toxic chemicals counted against CPTM’s Z-score ranking of chemicals, (**b**) the percentage of chemicals correctly identified as toxic at various Z-score cutoffs (e.g., top 100, 200, 300) after incorporating HNN predictions into the CPTM.

The chemicals were sorted in descending order based on the CPTM Z score. The top 300 ranked chemicals were annotated with the HNN-predicted toxic or non-toxic outcomes. We counted the instances where chemicals predicted to be toxic by the HNN (labeled as ‘1’) were correctly identified. The results, displayed in Figure 7a, illustrate the proportion of chemicals that the HNN correctly identified as toxic at various CPTM Z-score rankings. The percentage of chemicals correctly identified as toxic by the HNN at different Z-score cutoffs (e.g., top 100, 200, 300) remained consistent across the CPTM rankings for each LD_50_ threshold. Furthermore, the trend of correctly predicting experimental results improved with an increase in the LD_50_ threshold, with the highest accuracy of 65% being achieved at the 1500 mg/kg threshold. An increasing trend indicates that the CPTM is more effective at identifying toxic chemicals among those with higher Z scores. Conversely, a decreasing or fluctuating trend across different toxicity thresholds could indicate limitations in the model’s predictive ability. These findings again suggest that comparing the pathophysiological results predicted by the CPTM with those predicted by the HNN does not effectively demonstrate the CPTM’s efficacy in predicting chemical toxicity without integrating the machine-learning-based HNN results or vice versa. Consequently, we decided to incorporate the HNN-predicted outcomes with those predicted by the CPTM for further analysis.

*CPTM performance with HNN added*

We calculated a new Z score by adding one to the existing CPTM Z score for chemicals predicted to be toxic (assigned a value of one) by the HNN method. The chemicals were then re-sorted in descending order based on this new Z score, termed AI-CPTM (CPTM enhanced by ML predictions), where a higher value indicates higher toxicity. For the top 300 chemicals, we annotated the outcomes predicted by the HNN as either toxic or non-toxic, assigning a one for toxic and a zero for non-toxic. To determine the number of chemicals correctly predicted as toxic by the AI-CPTM among the top 100, 200, and 300, we counted the number of ‘1’s as indicating predictions of toxicity by the HNN in these subsets. The results are presented in Figure 7b. The trend of accurately predicting the HNN-predicted results improves with increasing LD_50_ thresholds, reaching the highest prediction accuracy. Interestingly, the correct prediction retrieval rate of AI-CPTM is 44% for an LD_50_ of 50 mg/kg, increases to 98% for 250 mg/kg, and achieves 100% for >500 mg/kg. The performance of AI-CPTM in correctly predicting the results (i.e., identifying toxic chemicals) varies among its top 100, 200, and 300 ranked chemicals at LD_50_ levels of 50 mg/kg, 250 mg/kg, 500 mg/kg, and 750 mg/kg, but not for 1500 mg/kg. This demonstrates a stark contrast to the trend of correct predictions by the CPTM alone.

In summary, the integration of machine-learning predictions significantly increases the number of correctly identified toxic chemicals among the top 100, 200, and 300 chemicals. This enhancement clearly demonstrates the benefits of including and augmenting the Z score with machine-learning insights to refine the predictive capabilities of the CPTM. The accuracy of identifying toxic chemicals within the newly ranked list of the AI-CPTM is superior when assessments are based on HNN predictions rather than solely on experimental outcomes. This improvement is attributed to the recalibrated Z score, by the HNN predictions, yielding a higher prediction rate than the original CPTM calculations. Additionally, in scenarios where the CPTM Z score is evaluated without the inclusion of HNN predictions, the overall trend indicates that the percentage of correctly predicted toxic chemicals, which is based on both the HNN-predicted and experimental outcomes, increases with an ascending LD_50_ (mg/kg) threshold (1500 > 750 > 500 > 250 > 50). However, with the integration of HNN predictions into the CPTM, the accuracy in predicting toxic chemicals based on both the HNN-predicted and the experimental data shows an increase up to the 500 mg/kg threshold and then plateaus. This plateau effect occurs because chemicals are considered toxic if their LD_50_ value is less than 500 mg/kg. Overall, AI-CPTM, which integrates the HNN with the CPTM, significantly improves the model’s predictive accuracy.

*II.2.*  *Chemical-Mixture Toxicity—Binary Classification*

In the process of applying AI-CPTM to chemical-mixture toxicity, we evaluated the performance of the AI-CPTM, which classifies virtual mixtures as either toxic or non-toxic. The virtual mixtures are created to compensate for the lack of data on experimental mixtures for the training set. These virtual mixtures were generated based on single-chemical oral LD_50_ data from rat and mouse studies listed in the ChemIDplus database. The training dataset comprised 3004 toxic and 3000 non-toxic virtual mixtures (refer to Materials and Methods for details). Additionally, we incorporated 182 mixtures sourced from the literature and 373 non-toxic drug combinations (details provided in Materials and Methods). In total, the training set included 3559 mixture combinations, with 3155 classified as toxic and 3404 as non-toxic. To assess toxicity, we considered the lowest effect level (LEL) chemical dose. We employed various LD_50_ thresholds for our analysis, ranging from 50 mg/kg to 1500 mg/kg, as described in our previous publications [45,47,48]. Toxicity predictions for 1050 chemically known toxic substances were conducted using both the HNN and CPTM methods. The HNN method categorizes chemicals as either toxic (assigned a value of one) or non-toxic (assigned a value of zero), whereas the CPTM method outputs a Z score. These combined Z scores (AI-CPTM) are then rank-ordered, with higher values indicating greater toxicity. A more detailed explanation of the descriptor calculation and the toxicity prediction process for mixtures using the HNN can be found in the Materials and Methods Section, as well as in our published studies [45,47,48]. Additionally, the methodology for calculating the CPTM Z score is thoroughly detailed in our earlier publications [49].

II.2.1. Accuracy Based on Experimental Toxicity

We conducted a comparative analysis to assess whether incorporating HNN predictions enhances the CPTM performance in predicting chemical-mixture toxicity. This involved evaluating the performance of individual CPTM predictions and combined CPTM + HNN (AI-CPTM) predictions against 182 mixed chemicals already known to be toxic from experimental studies. This targeted analysis determines the accuracy of experimentally determined toxic chemical mixtures when ranked according to the CPTM Z score, both with and without the inclusion of HNN predictions, as described below.

*CPTM Performance without HNN Predictions*

**Figure 8:**
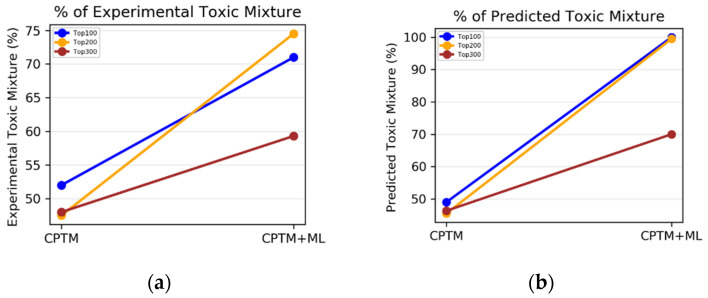
(**a**) Percentage of experimental 1s (toxic) counted against CPTM’s Z-score ranking and ranking obtained after machine-learning (ML) predictions integrated into the Z score. (**b**) The percentage of predicted 1s counted against CPTM’s Z-score ranking, and the ranking was obtained after machine-learning (ML) predictions were integrated into the Z score.

Chemical mixtures were ranked in descending order based on their CPTM Z scores. We then annotated the experimentally assessed toxic or non-toxic outcomes for the top 300 chemical mixtures, assigning a ‘1’ for toxic and a ‘0’ for non-toxic. To evaluate the accuracy of the CPTM predictions among the top 300 mixtures, we counted the number of ‘1’s known to be toxic from experimental studies. The results, displayed in Figure 8a, show the percentage of experimentally determined toxic chemical mixtures ranked according to the CPTM Z score.

*CPTM Performance with HNN Added (AI-CPTM)*

Next, new Z scores were generated by adding a value of one to the original CPTM Z scores for mixtures predicted as toxic by HNN. The efficacy of the AI-CPTM was then assessed by counting the predicted toxic mixtures among the top 100, 200, and 300. Similar to that discussed above, we annotated the experimentally assessed outcomes for the top 300 mixtures as toxic (‘1’) or non-toxic (‘0’), presuming that the top 300 chemicals are relatively toxic. To determine the number of correctly predicted chemicals by the AI-CPTM in the top 100, 200, and 300, we matched the ‘1’s known to be toxic from experimental studies. These findings, displayed in Figure 8a, show the percentage of experimentally determined toxic chemicals as per the AI-CPTM’s Z-score ranking. Figure 8a reveals that the percentage of mixtures correctly identified as toxic at various Z-score cutoffs (e.g., top 100, 200, 300) improved after incorporating HNN predictions into the CPTM. Specifically, the AI-CPTM performance in accurately predicting experimental results (i.e., toxic chemicals) varies across the top 100, 200, and 300 ranked chemical mixtures, with the top 100 and 200 mixtures showing higher predictive performance. For the top 100 and 200, the AI-CPTM predictive accuracy increased dramatically, from 50% to 75%, while for the top 300, the performance reached 60%. This increasing trend suggests that the AI-CPTM is more proficient at identifying toxic chemicals among those with higher Z scores.

II.2.2. Accuracy Based on HNN Predicted Toxicity

Next, we carried out the analysis that involved evaluating the performance of standalone CPTM predictions against those combined with HNN predictions (AI-CPTM), using the actual HNN-alone predictions for 3155 toxic chemical mixtures as a benchmark described below.

*CPTM Performance without HNN Predictions*

To determine the number of correctly predicted chemical mixtures by the CPTM within the top 100, 200, and 300, we counted the ‘1’s predicted to be toxic by the HNN. The results in Figure 8b illustrate the percentage of HNN-predicted toxic chemical mixtures as per the CPTM’s Z-score ranking. Figure 8b shows the performance of CPTM in correctly identifying toxic chemical mixtures at various Z-score cutoffs (e.g., top 100, 200, 300). The CPTM capability to accurately predict HNN-identified toxic outcomes (i.e., ‘1s’—toxic chemicals) remained consistent across the top 100, 200, and 300, standing at 49%. This medium predictive trend may indicate limitations in the model’s predictive capacity, establishing a baseline for the effectiveness of the CPTM in predicting chemical-mixture toxicity without machine-learning HNN enhancements.

*CPTM Performance with HNN Added (AI-CPTM)*

The efficacy of AI-CPTM was evaluated by counting the predicted toxic mixtures among the top 100, 200, and 300. The same annotation process for the HNN-predicted toxic or non-toxic outcomes was applied to the top 300 mixtures. The number of correctly predicted chemicals by the AI-CPTM in the top 100, 200, and 300 was determined by matching the ‘1’s predicted to be toxic by the HNN (Figure 8b). Figure 8b reveals that after incorporating the HNN predictions into the CPTM, the percentage of mixtures correctly identified as toxic at various Z-score cutoffs (e.g., top 100, 200, 300) increased significantly. The AI-CPTM performance in accurately predicting toxic mixtures varied across the top 200 and 300 ranked mixtures, with the top 100 and 200 achieving the highest predictive performance at 99%. This marked an improvement from 49% to 99% for the top 100 and 200, and an increase to 70% for the top 300, suggesting the AI-CPTM enhanced accuracy for identifying toxic chemicals, particularly among those with higher Z scores. The integration of HNN predictions into the CPTM signifies an enhanced model predictive capability for chemical-mixture toxicity.

In summary, in the context of the accuracy calculated based on experimental toxicity, the integration of HNN with the CPTM has significantly increased the number of correctly identified toxic mixtures within the top 100, 200, and 300 mixtures. This enhancement emphasizes the potential of improving the CPTM model’s predictive accuracy through machine-learning predictions. Notably, the AI-CPTM’s ability to accurately classify toxic chemical mixtures in its newly ranked list was superior when based on HNN-predicted outcomes compared to actual experimental outcomes. This improvement comes from the recalibrated Z score, which is based on the HNN predictions and shows a higher prediction rate than the CPTM alone. Similarly, in the case of accuracy calculated based on HNN-predicted toxicity, the findings demonstrate that the incorporation of HNN predictions into the CPTM noticeably enhances the model’s capability to predict chemical-mixture toxicity. By integrating machine learning, the AI-CPTM delivers more refined and accurate toxicity predictions, potentially identifying subtle patterns and correlations that are not apparent with the HNN alone or CPTM alone. However, it is important to recognize the complexities and potential biases inherent in machine-learning algorithms, which could influence the interpretation and generalizability of the outcomes. This challenge is offset by the pathophysiological insights offered by the CPTM model, emphasizing the significant advancements in toxicity prediction achieved by integrating HNN.

*II.3.*  *Experimental Validation*

**Figure 9:**
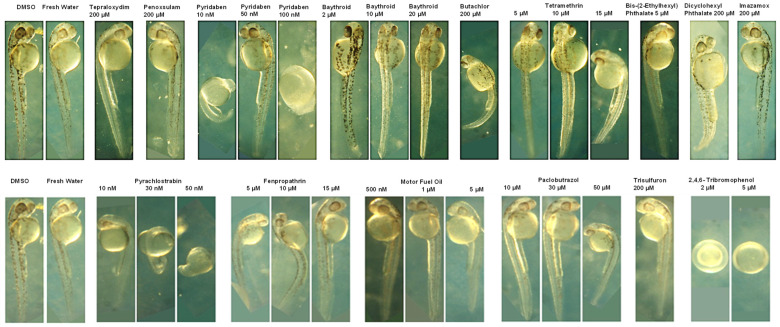
Dose-dependent toxicity assessment of AI-CPTM predicted chemicals (Table 1, Table 2, Table 3 and Table 4) as the lowest dose leading to, what is defined here, as significant developmental abnormalities at 2 days post-fertilization (2 dpf) in a dose-response zebrafish live assay.

**Figure 10:**
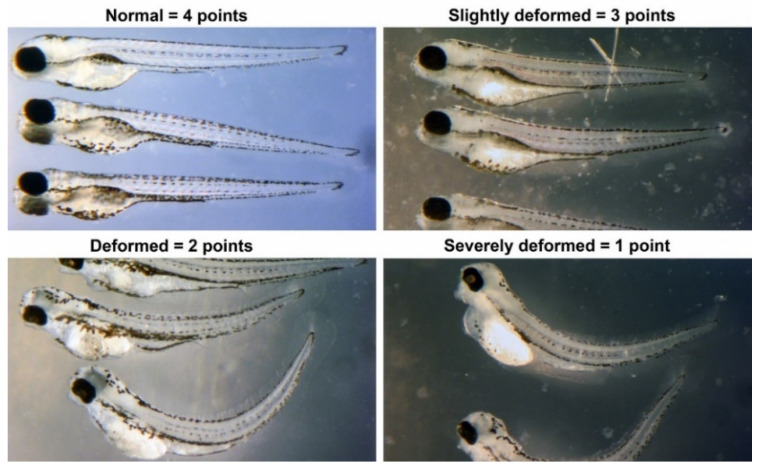
Mixed chemical phenotypes and how they are scored. The mixed chemical phenotype score is the sum of the scores for the five individual fish in a well. Two wells were treated per chemical combination, and the scores are averaged for the wells. Scoring was as follows: dead = 0; severely deformed = 1; deformed = 2; slightly deformed = 3; and normal phenotypes = 4.

**Figure 11:**
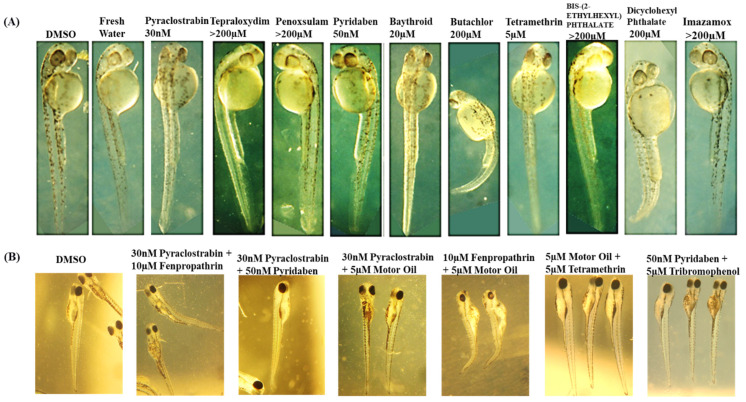
The results of zebrafish toxicity studies for panel (**A**) selected single chemicals and panel (**B**) their mixture combinations. Panel A is the toxicity data obtained from single-chemical dose-response studies that were used to inform the mixed chemical toxicity studies. For panel B, the mixture toxicity were assessed based on the presence of significant developmental abnormalities in the embryos, with DMSO and water as controls. The outcomes regarding toxicity, developmental abnormalities, and chemical–chemical interactions are in Table 3 and Figure 9 and Figure 10. The rationale behind the selection of doses for mixture toxicity studies is detailed within the main text.

In a dose-response zebrafish-embryo toxicity assay, we assessed the AI-CPTM-predicted 11 toxic chemicals, including PFAS (Table 1 and Figure 9, Figure 10 and Figure 11). We used the zebrafish embryos as a model for determining the potential hazards posed by the chemicals to humans apart from aquatic life. Further, these zebrafish experiments were used to establish the toxic concentrations for each chemical. Subsequently, we created 38 binary mixtures from these 11 chemicals. In the iterative simulations, these determined concentrations were used to calculate the dose-dependent ratios and molecular descriptors of these 11 component chemicals within each mixture (Table 2). These descriptors were subsequently employed as input features for the AI-CPTM. The toxicity assessments of these mixtures were computed using AI-CPTM, and the results of these evaluations are presented in Table 2 and Table 3, including the corresponding toxicity outcomes determined from the zebrafish experiments (Figure 10 and Figure 11). This comprehensive experimental analysis validated the AI-CPTM’s predictive performance for both single chemicals and their mixtures, and its ability to understand the mixture effects of chemical combinations, including PFAS. Consequently, it enhanced the predictive capability of the AI-CPTM for mixed toxicological evaluations. The detailed results are discussed in the below sections.

II.3.1. Zebrafish-embryo toxicity studies of single chemicals

Dose-dependent toxicities in zebrafish embryos were assessed by the lowest dose that caused significant developmental abnormalities 2 days post-fertilization (2 dpf) in a dose-response live assay. The results, displayed in Figure 9, Table 3, and Supplementary Material S2, detail the deformities observed in the embryos as indicators of chemical toxicity. We used DMSO and fresh water as controls for comparing the effects of each chemical and confirmed that the observed effects in the embryos were due to the chemicals tested. Starting with Pyraclostrobin, it was identified as toxic at 30 nM. It induced developmental delays in the embryos without visible deformities. However, increasing the concentration to 100 nM resulted in embryo lysis, demonstrating an apparent dose-dependent toxicity. This pattern suggests that Pyraclostrobin may interfere with essential developmental processes at a cellular level. Fenpropathrin exhibited toxicity at 10 µM, resulting in slight developmental delays and dorsal arching, with a twitching phenotype observed by 2 dpf at both 10 µM and 15 µM. It appeared relatively normal at 5 µM. This twitching phenotype indicates specific neuronal toxicity. Motor fuel oil showed toxicity at 5 µM, characterized by a delayed onset (around 36 hpf), slight ventral curvature, and skinniness at 5 µM, but was relatively normal at 1 µM, with 100% lethality at 10 µM. The symptoms’ delayed onset suggests that the motor fuel oil’s toxic effects might involve pathways activated later in the developmental process. Paclobutrazol at 50 µM also showed severe effects, including pronounced ventral curvature and cardiac edema, indicating its potent impact on cardiac development and overall embryonic growth. These symptoms were absent at lower concentrations (30 µM), highlighting a dosage-sensitive relationship.

In contrast, Triasulfuron, Tepraloxydim, and Penoxsulam did not exhibit toxicity even at high concentrations (200 µM), suggesting that their modes of action may not be active in zebrafish embryogenesis within the tested range. 2,4,6-Tribromophenol was particularly potent, stalling development as early as the 2-somite stage at concentrations as low as 2 µM and was lethal by 10 µM. This indicates a strong embryotoxic effect, interfering with very early developmental stages. Pyridaben was found to be toxic at 50 nM, causing developmental stalling at the 8-somite stage at 100 nM, affecting developmental progression (32 hpf), and inducing morphological abnormalities in the hindbrain region. This suggests disruptions in neural development characterized by a slightly elongated and thin hindbrain region at 50 nM. For Baythroid, although toxic at 20 µM, variable phenotypes, with issues such as solubility and observed precipitation, complicate the understanding of its direct effects on embryo development. Butachlor was toxic at 200 µM, leading to significant ventral curvature and cardiac edema, suggesting severe developmental toxicity, and was normal at 100 µM.

Conversely, Bis-(2-Ethylhexyl) Phthalate showed no toxic effects up to 200 µM, indicating a lack of detrimental interaction with the zebrafish developmental pathways at these concentrations. Both Tetramethrin and Dicyclohexyl Phthalate showed toxicity at 5 µM and 200 µM, respectively, with specific developmental delays and morphological abnormalities, indicating that these chemicals could disrupt normal embryonic development even at low concentrations. Finally, Imazamox appeared to be non-toxic up to 200 µM, possibly due to its inability to interfere with the pathways that are essential for early development in zebrafish.

II.3.2. Zebrafish-Embryo Toxicity of Chemical Mixtures and Chemical Interactions Analysis

*II.3.2.A.*  *Measurement of Mixture Toxicity in Zebrafish Models*

*Approach for Assessing Chemical Mixtures in Zebrafish*

First, the toxicity of single chemicals in zebrafish embryos is determined. Using the toxicity data of single chemicals as a baseline, we screened mixtures for altered activity and characterized the optimal concentration ratios of components. For chemical treatments, 1000× working stocks are prepared in DMSO. Dechorionated zebrafish embryos, eight per well, are placed in 0.5 mL of fish water (0.3 g/L sea salt) in 24-well plates. The 1000× stock solution of chemicals is diluted to 2× in fish water with 2% DMSO added. Then, 0.5 mL of the 2× diluted chemical solution is added to the 0.5 mL of fish water containing the embryos to achieve a final concentration of 1× compound in 1% DMSO.

*Measurement of Developmental Toxicity*

We assessed individual chemicals for developmental toxicity, indicating any disruption in embryonic development from 1 to 4 days post-fertilization (dpf). Embryos are exposed to chemicals in their bath water from 1 dpf to 4 dpf, without changing the water during exposure. Initially, chemicals are screened by testing them at a series of concentrations increasing by half-log steps. Teratogenicity is quantified based on the severity of developmental abnormalities, as shown in Figure 9, Figure 10 and Figure 11. Each embryo is assigned a score reflecting the severity of observed phenotypes, with the average score representing the group. The EC_50_ for developmental toxicity is defined as a score of five on this teratogenicity scale (Figure 10 and Supplementary Material S2). Once the effective range is identified, the EC_50_ is determined by testing a linear series of concentrations spanning from non-toxic to toxic levels. For chemicals not toxic at 10 µM, tests are extended up to 200 µM, with those showing no effects at this level considered non-toxic.

A thorough evaluation is conducted on how various chemicals and their combinations affect the development of zebrafish embryos in terms of toxicity, as shown in Figure 11. Pyraclostrobin is examined across a range from 30 nm to 200 µm, showing minimal effects at the lowest concentration but significant embryonic damage at 200 µm, highlighting a clear dose-dependent increase in toxicity. The herbicides terapolyxylim and penosulam, both administered at 200 µm, cause moderate deformities, like slight curvature of the spine, indicating a disruption in developmental processes. Pyridaben, at a lower concentration of 50 nm, already leads to noticeable developmental delays and morphological abnormalities, emphasizing the potential for significant impact even at lower doses. Baythroid and butachlor, as well as tetramethrin and phthalate, at 200 µm, induce pronounced deformities, such as severe spinal curvatures and edema, suggesting strong toxic impacts that disrupt physiological development. The most severe effects are observed with diclobenyl phthalate and imazamox at 200 µm, where exposure results in marked malformations and growth retardation, highlighting their potent embryotoxic effects. Analyzing the toxicity of chemical mixtures, which better reflects realistic environmental exposures, with non-toxic DMSO as a control, shows embryos with normal development, compared to the observed effects in chemical mixture treated groups. The mixture of pyraclostrobin at 30 nm with 10 µm fenpropathrin exhibits synergistic effects, leading to more severe developmental defects than either chemical alone, suggesting amplified toxicity. Further, mixtures of pyraclostrobin with motor oil or tertramethrin and the combination of fenpropathrin with motor fuel oil at 10 µm and 5 µm, respectively, show extremely detrimental effects on embryo development, with significant abnormalities, such as severe curvature and edema. These findings indicate that certain combinations can severely disrupt embryonic development. Moreover, the combination of pyridaben at 50 nm with trimethobenzol at 5 µm results in a significant increase in toxicity, causing extensive developmental deformities. This comprehensive analysis emphasizes the need for a detailed consideration of both the individual and combined effects of chemicals in environmental and regulatory contexts. Further, the chemical interaction analysis is discussed in detail in the section below.

**III.1.**  **Comparison of zebrafish toxicity outcomes with the results from machine-learning models for chemical mixtures**

We tested the top 300 chemical mixtures, as prioritized by AI-CPTM, which were randomly selected and readily purchasable, on zebrafish embryos. Based on the degree of deformity, morphological changes, and survival rate of the embryos (Figure 9, Figure 10 and Figure 11), we classified each binary mixture as toxic (‘1’) or non-toxic (‘0’) (Table 2). We then compared the toxicity predictions made by the machine-learning model with the results obtained from the zebrafish assays. Table 3 summarizes the prediction outcomes using AI-CPTM, HNN, RF, bagging, and AdaBoost methods, alongside the zebrafish-determined toxicity, with ‘1’ indicating toxic and ‘0’ indicating non-toxic. Our AI-CPTM method achieved an accuracy of 81% in predicting the zebrafish mixture toxicity outcomes, followed by our standalone AI-HNN at 70.2%, random forest at 62.1%, bagging at 54%, and AdaBoost at 51.3% (Table 2). The predictive capability of AI-CPTM for chemical-mixture toxicity is observed to be superior to that of HNN alone and the other machine-learning methods tested. Our standalone AI-HNN method also surpassed the other machine-learning techniques in predictive performance (Table 2). Notably, the predictions from AI-CPTM agree with the experimental chemical toxicity outcomes observed in zebrafish. We anticipate an improved trend in the prediction accuracy for chemical-mixture toxicity, as we further refine and optimize the AI-CPTM method with additional concentration and dose-response data. This extensive validation exercise highlights each predictive approach’s relative strengths and weaknesses in different chemical interaction scenarios.

*III 1.1.*  *Determination of EC_50_ and Measurement of Mixed Chemical Interactions*

The EC_50_, indicating the median effective concentration for developmental toxicity, is determined using morphological criteria. Fish are identified by date and well number (Figure 10). In each well containing five fish, the mixed compound phenotype score is the sum of the scores for the individual fish. Two wells are treated per combination, and the scores are averaged across these wells (Supplementary Material S2). Scoring follows this scale: dead = 0; severely deformed = 1; deformed = 2; slightly deformed = 3; normal phenotypes = 4. Chemical interactions in mixtures are classified as synergistic, additive, no interaction, inconclusive, or antagonistic based on the comparative toxicity of the mixtures versus the individual components. For example, if compound #1 scores 17 and compound #2 scores 15, a combined average score of 15 indicates no interaction, suggesting that compound #1’s toxicity does not exacerbate that of compound #2 (Supplementary Material S2). The EC_50_ and lowest observable effect concentration (LOEC) for binary mixtures are determined by testing a linear concentration series of the first chemical against five concentrations of the second chemical, including a vehicle control.

*III 1.2.*  *Comparison of Predicted vs. Experimental Mixed Chemical Interactions*

Focusing on specific combinations (Table 2 and Figure 11), such as Pyraclostrobin at 0.01 µM mixed with Fenpropathrin at 5 µM, we observe that while RF, bagging, and AdaBoost predicted toxicity, both AI-CPTM and HNN did not predict any harmful interaction. The experimental results supported the predictions of AI-CPTM and HNN, showing no interaction, thus validating these models as more accurate in this scenario. This pattern is consistent in other mixtures involving Pyraclostrobin with different chemicals, such as motor fuel oil, where again, AI-CPTM and HNN correctly predicted no interaction while the other models did not. In contrast, the combination of Fenpropathrin and fuel oil, where most models except AI-HNN anticipated an additive effect, aligns with the experimental observations of increased toxicity. This scenario validates the predictions of RF, bagging, and AdaBoost, emphasizing their utility in identifying potential additive interactions. Investigating broader interactions involving chemicals such as Paclobutrazol, 2,4,6-Tribromophenol, and Pyridaben reveals a trend where AI-CPTM generally predicts potential toxicity. The mixtures involving Butachlor, Tetramethrin, and Dicyclohexyl Phthalate further demonstrate the predictive power of the AI-CPTM and HNN models. Many predictions of synergistic interactions by these models were confirmed by the experimental results, particularly when the models were in agreement. This consistency highlights the effectiveness of AI-CPTM for scenarios expecting significant chemical interactions. A distinct case is the mixture of Perfluorooctane Sulfonic Acid (PFOS) and Perfluorooctanoic Acid (PFOA), where all models predicted a synergistic interaction, which was robustly validated by the experimental findings discussed in below sections. Taken together, the overall analysis indicates that AI-CPTM and HNN are particularly reliable not only for predicting chemical mixture toxicity but also for correctly predicting a mixture’s chemical interaction effects. The RF, bagging, and AdaBoost models perform well in most cases, where chemical interactions enhance toxicity, i.e., synergistic effects. Finally, these findings emphasize the importance of studying chemical interactions for environmental safety assessments, as these mixtures can lead to unexpected and often more severe biological or health impacts.

**Table 2:** The toxicity predictions for binary mixtures of ten chemicals, as detailed in Table 2, using four machine-learning methods: hybrid neural network (HNN), random forest (RF), bagging, and AdaBoost. The table also includes the concentrations of the components in each binary mixture. The last two columns provide experimental toxicity and chemical interaction effects that support a reference to evaluate the accuracy of the machine-learning models.

Chemical 1	Concentration LC_50_ (µM)	Chemical 2	Concentration LC_50_ (µM)	AI-CPTM	AI-HNN	RF	Bagging	Adaboost	Experiment (Zebrafish)	Mixture Chemical Interaction
Pyraclostrobin	0.01	Fenpropathrin	5	0	0	1	1	1	0	no interaction
		Alpha, alpha’-(1-methylethylenediimino)di-ortho-cresol (motor fuel oil)	2.5	0	0	1	1	1	0	no interaction
		Paclobutrazol	50	1	1	1	1	1	0	inconclusive
		2,4,6-Tribromophenol	2	1	1	1	1	1	1	inconclusive
		Pyridaben	0.05	0	1	1	1	1	0	inconclusive
		Butachlor	100	1	1	0	0	0	1	no interaction
		Tetramethrin	5	1	0	0	1	1	1	inconclusive
		Dicyclohexyl Phthalate (DCHP)	100	1	0	1	1	0	1	inconclusive
Fenpropathrin	5	Alpha, alpha’-(1-methylethylenediimino)di-ortho-cresol	2.5	1	1	1	0	1	1	additive
		Paclobutrazol	50	1	1	1	1	1	1	additive
		2,4,6-Tribromophenol	2	1	1	1	1	1	1	additive
		Pyridaben Pestanal	0.05	1	1	1	1	1	0	no interaction
		Butachlor	100	1	1	0	0	0	1	additive
		Tetramethrin	5	1	1	1	1	1	1	synergistic
		Dicyclohexyl Phthalate (DCHP)	100	1	1	1	1	0	1	synergistic
Alpha, alpha’-(1-methylethylenediimino)di-ortho-cresol (motor oil)	2.5	Paclobutrazol	50	1	1	1	1	1	1	additive
		2,4,6-Tribromophenol	2	1	1	1	1	1	1	additive
		Pyridaben Pestanal	0.05	0	0	0	1	1	0	inconclusive
		Butachlor	100	0	0	1	0	0	1	additive
		Tetramethrin	5	0	0	0	0	1	0	no interaction
		Dicyclohexyl Phthalate (DCHP)	100	0	0	0	0	0	0	no interaction
Paclobutrazol	50	2,4,6-Tribromophenol	2	0	0	1	1	1	0	no interaction
		Pyridaben	0.05	1	1	1	1	1	1	additive
		Butachlor	100	1	1	1	1	1	1	synergistic
		Tetramethrin	5	0	0	1	0	1	0	additive
		Dicyclohexyl Phthalate (DCHP)	100	1	1	1	0	1	0	additive
2,4,6 -Tribromophenol	2	Pyridaben Pestanal	0.05	1	1	1	1	1	0	no interaction
		Butachlor	100	1	1	0	0	0	1	synergistic
		Tetramethrin	5	1	1	1	1	1	1	additive
		Dicyclohexyl Phthalate (DCHP)	100	1	1	1	0	0	1	no interaction
Pyridaben	0.05	Butachlor	100	1	1	1	1	1	1	synergistic
		Tetramethrin	5	1	1	1	1	0	0	inconclusive
		Dicyclohexyl Phthalate (DCHP)	100	1	1	1	1	0	0	inconclusive
Butachlor	100	Tetramethrin	5	1	1	1	0	1	1	synergistic
		Dicyclohexyl Phthalate (DCHP)	100	1	1	1	1	1	1	synergistic
Tetramethrin	5	Dicyclohexyl Phthalate (DCHP)	100	1	1	1	1	1	1	inconclusive
Perfluorooctane sulfonic acid (PFOS)	53	Perfluorooctanoic acid (PFOA)	187.5	1	1	1	1	1	1	synergistic

**III.2.**  **Toxicity Studies of Perfluorooctane Sulfonic Acid (PFOS) and Perfluorooctanoic Acid (PFOA) chemicals and their mixtures on Zebrafish Embryos**

**Figure 12:**
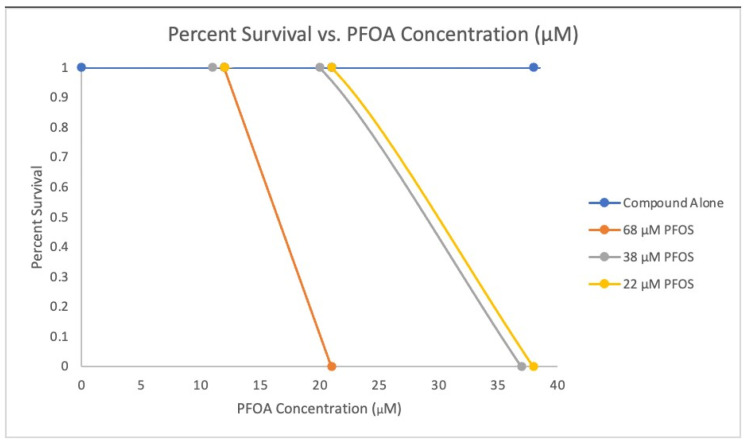
Dose-response of zebrafish survival rates upon exposure to PFOA and PFOS mixtures (Table 4).

The methodology for assessing the toxicity in zebrafish is detailed in our earlier publication [49]. The data presented in Table 3 and Table 4 and Figure 12 show the acute and sub-lethal toxicity of PFOS and PFOA individually and in combination with zebrafish embryos. This initial study offered a comparative insight into their toxic profiles in terms of lethal (LD_50_) and effective (EC_50_) concentrations, which are important for measuring their toxic effects. As indicated in Table 3 and Table 4 and Figure 12, the lethal dose (LD_50_) and the effective concentration (EC_50_) for PFOS have been determined to be 53 µM and 11 µM, respectively. This indicates a high level of acute toxicity, making PFOS notably hazardous. For PFOA, these values are 187.5 µM for LD_50_ and 29.5 µM for EC_50_. These concentrations reflect the exposure of zebrafish embryos to PFOS and PFOA at 2 days post-fertilization (dpf) for one day at a temperature of 33 °C. Further, in the case of PFOA, at 187.5 µM, the LD_50_ concentration for PFOA is significantly higher than that for PFOS. This suggests that, under similar conditions, PFOA is less lethal to zebrafish embryos, though still toxic. The effective concentrations (EC_50_) of PFOS, at 11 µM, induce sub-lethal effects in half of the zebrafish-embryo population. These effects include severe developmental abnormalities, such as bent body axis, edema, cloudy tissue formation, and developmental delays, signaling significant toxicity even at lower concentrations. Whereas PFOA requires a higher concentration of 29.5 µM to achieve similar sub-lethal effects. This still presents considerable toxicity, indicating that, while PFOA may be less acutely toxic than PFOS, it has the ability to cause developmental disruption at higher concentrations.

**Table 3:** Toxicity of Perfluorooctane Sulfonic acid (PFOS) and perfluorooctanoic acid (PFOA) in zebrafish embryos. The toxicity of two chemicals, PFOS and PFOA, in zebrafish embryos and their effects at critical early development stages. Displayed are the median lethal dose (LD₅₀) and the median effective concentration (EC₅₀) for embryos exposed to these substances at 2 days post-fertilization. This exposure occurs over a 24 h period at a controlled temperature of 33 °C. The LD₅₀ represents the concentrations of PFOS or PFOA required to cause death in 50% of the zebrafish embryos within the exposure timeframe. The EC₅₀ measures the concentration at which 50% of the embryos show sub-lethal but adverse effects. These effects include a range of developmental abnormalities, such as bent body axis, which indicates spinal or skeletal malformations; edema; and cloudy tissue, which may suggest cellular damage or necrosis and developmental delays and are observable as slowed growth or delayed milestone achievement compared to unexposed embryos.

Compound	LD_50_	EC_50_
**PFOS**	53 µM	11 µM
**PFOA**	187.5 µM	29.5 µM

**Table 4:** The LD₅₀ concentrations for mixtures of PFOS and PFOA. The LD₅₀ for PFOA, when combined with three different concentrations of PFOS, is determined to understand how the presence of PFOS affects the toxicity of PFOA and the chemical interaction effects under various exposure conditions.

**Concentration of PFOS Present** (µM)	68 µM PFOS	38 µM PFOS	22 µM PFOS
**LD50 (added PFOA concentration** (µM)	16.5 µM PFOA	29 µM PFOA	29.5 µM PFOA

*III. 2.1.*  *Zebrafish Survival upon exposure to PFOA and PFOS mixtures*

PFOS and PFOA Mixture Zebrafish Survival Assay

We explored further the combined effects of PFOS and PFOA, as shown in Table 4 and Figure 12, to examine how varying concentrations of PFOS affect the LD_50_ of PFOA in a mixture. The LD_50_ is the dose at which 50% of the embryos are dead. In comparison, the EC50 is the dose at which 50% of the embryos exhibit any form of abnormality, including a bent body axis, edema, cloudy tissue, and developmental delay. A five dose-response curve for PFOA was tested against three fixed concentrations, 68 µM, 38 µM, and 22 µM of PFOS at 2 dpf for one day at 33 °C. These preliminary dose curves demonstrate variability with the zebrafish assay, as shown by the calculated LD_50_ value of 53 µM PFOS (Table 4) versus 100% survival at 68 µM PFAS for the experiment presented in Figure 12. Nonetheless, as each experiment is internally controlled, relative toxicity and mixture interactions are accurately detected. The combination of 68 µM PFOS and 16.5 µM PFOA shows that a lower concentration of PFOA is needed to reach LD_50_ when PFOS is at a higher concentration. The combination of 38 µM PFOS and 29 µM PFOA shows an increase in the required LD_50_ concentration of PFOA as the PFOS level is reduced, whereas the combination of 22 µM PFOS and 29.5 µM PFOA showed even with a further reduction in PFOS, the LD_50_ of PFOA remains similar to that in the 38 µM PFOS scenario. These findings suggest that higher PFOS concentrations significantly decrease the PFOA concentration required to lethally affect 50% of the zebrafish embryos. This interaction pattern between PFOS and PFOA indicates a synergistic effect, where the toxicity of one chemical is enhanced by the presence of another (Table 2). Taken together (Table 3 and Table 4 and Figure 12), these data provide standalone and combinatory PFOS and PFOA toxicity effects.

Further, the comparison between the LD_50_ and EC_50_ values for PFOS and PFOA clearly shows that PFOS is more toxic than PFOA to zebrafish embryos. PFOS requires a lower concentration to be lethal and affects development at much lower concentrations compared to PFOA. It is noted that the single-compound LD50/EC50 values are the average of three independent experiments, while the mixture dose-response results are taken from a single experiment. One-hundred percent survival at 68 μM versus an LD50 of 53 μM is within the variability of these experiments and is, thus, not particularly concerning in this context. This is unlikely to be an antagonistic effect, as 100% survival at 68 μM PFOS was seen at 0 μM PFOA. Five concentrations of PFOA were tested to generate the dose-response curves. Furthermore, Figure 12 shows low precision curves, although the ranges and suggested interaction between PFOA and PFOS are accurate based on these data. Overall, these experiments support predictions of AI-CPTM toxicity and chemical interaction effects.

## 4. Limitations

This study leverages a machine-learning-driven pathophysiology method to evaluate the dose-dependent toxicity of hazardous chemical mixtures, incorporating experimental validations using zebrafish-embryo assays. Despite achieving promising results, several limitations and areas for future research should be addressed to enhance the model’s applicability and reliability. First, although the data set is extensive, it relies significantly on virtual mixtures to address the lack of empirical data. This dependency on simulated data introduces potential biases and may limit the generalizability of the findings. While the dataset contains 981 experimental mixtures enriched with virtual mixtures, it does not capture the full spectrum of environmental mixtures, particularly those involving complex mixers of environmental compounds. This limitation adds potential biases in the AI-HNN model, as it might not fully represent the diverse chemicals and their interactions encountered in real-world scenarios. Despite their high accuracy, machine-learning algorithms are susceptible to several biases that can affect the reliability of toxicity predictions. One key type is sampling bias, which occurs if the training data do not adequately represent the diversity of real-world chemical mixtures. This can lead to models that perform well on the training data but fail to generalize to new, hidden data. Another critical issue is label bias, where inaccuracies or inconsistencies in the labeled data used for training can skew the model’s learning process, leading to inaccurate predictions.

Furthermore, algorithmic bias may arise from how machine-learning algorithms prioritize certain features or patterns over others, potentially ignoring significant but less frequent interactions. These biases can influence the interpretation and generalizability of the results, stressing the need for rigorous validation and refinement of the models. Further, the CPTM pathophysiology model does not fully capture the toxicokinetics, toxicodynamics, and exposomics of chemical mixtures.

Additionally, while zebrafish-embryo assays provide valuable insights into developmental toxicity, they do not fully replicate human biological responses. This limitation restricts the direct applicability of these findings to human health risk assessments. The absence of human models in the validation phase further complicates this issue, as it is important to confirm the relevance of toxicity predictions to human health. Moreover, while the study addressed additive, synergistic, independent, and antagonistic interactions among chemicals, it did not explore all possible interactions, particularly those involving complex mixtures of more than two chemicals. This gap in the analysis means that the model may not fully account for the comprehensive nature of chemical interactions in the environment.

## 5. Future Studies

To address these limitations, our future research will focus on expanding the dataset with more empirical data from diverse environmental and industrial sources, specifically targeting emerging contaminants, such as PFAS and nanoplastics, to improve the accuracy, robustness, and generalizability of the models for the de novo data. We will also enhance our machine-learning algorithms that reduce biases and improve the interpretability of toxicity predictions. This will be complemented by implementing rigorous cross-validation techniques, including external validations with independent and de novo datasets. We are extending the approach to study more complex mixtures involving multiple chemicals that will provide deeper insights into their combined or cumulative effects. The pathophysiology method is expanded by including more advanced toxicokinetics, a toxicodynamics pipeline with exposomics, and epidemiological data.

Further improvements will include identifying potential biomarkers and predictive indicators of chemical toxicity to aid in early detection and intervention strategies. We are incorporating human-cell-based assays, including mechanistic studies, to understand the biological pathways and molecular interactions underlying the observed toxic effects. This would enhance the relevance of the findings to human health risk assessments, providing more accurate representations of human biological responses to chemical exposures. The continued development and refinement of AI-CPTM will be essential for improving our ability to predict the toxic and cancer risks posed by hazardous chemical mixtures, ultimately contributing to better public health outcomes.

## 6. Conclusions

In this study, to predict the dose-dependent toxicity of chemicals and their mixtures, we carried out studies in three primary phases. In the first phase, we deployed machine-learning models for initial toxicity prediction. In the second phase, we carried out the subsequent integration HNN method with the physiologically based method to enhance predictive accuracy and reliability. In the final phase, we performed statistical, literature, and experimental validation studies. In the beginning, we developed and tested multiple machine-learning models, including binary and multiclass classification and regression models based on our HNN method, and several other machine-learning methods such as RF, bagging, AdaBoost, SVR, GB, KR, DT, and KN. The dataset comprised 981 experimental mixtures and was expanded with virtual mixtures to address the lack of empirical data. We also employed an ensemble of these methods to arrive at consensus scores to assess their predictive capabilities. Detailed statistical analyses were performed to validate the results of the models and evaluate reliability and rigor through stratified 10-fold cross-validation and multiple iterations. Performance metrics, including accuracy, sensitivity, specificity, precision, and AUC, were analyzed. Our HNN models achieved comparatively high accuracies, with AUC values exceeding 90%, demonstrating robust predictive capabilities. Further, we introduced a novel methodology, AI-CPTM, which integrates an HNN with our pathophysiology method, CPTM, for the dose-dependent assessment of hazardous chemical mixtures. The AI-CPTM integrative approach leverages the predictive power of machine learning together with the toxicodynamics, toxicokinetics, and pathophysiological insights provided by CPTM.

Integrating HNN predictions with CPTM (AI-CPTM) substantially increased the number of correctly identified toxic chemical mixtures. The AI-CPTM method demonstrated superior accuracy in predicting toxicity for the top 100, 200, and 300 ranked mixtures, with performance improvements from 49% to 99% in some cases. This enhancement underlines the value of combining machine-learning insights with pathophysiological data to refine toxicity predictions. Experimental validation using zebrafish-embryo assays confirmed the predictive capabilities of AI-CPTM. The method achieved 81% accuracy in predicting toxicity compared to experimental outcomes, outperforming other ML techniques. The AI-CPTM experimental validations with zebrafish-embryo toxicity assays provided a biological confirmation and highlighted its potential for identifying toxic effects at various developmental stages, which are critical endpoints in toxicological studies. These rigorous validations, including zebrafish-embryo toxicity assays and AI-CPTM, have shown substantial improvements in identifying toxic chemical mixtures compared to traditional methods. This validation emphasizes the practical applicability of AI-CPTM in real-world toxicological assessments of chemicals and their mixtures. We are now expanding the applicability of AI-CPTM to predict and validate the carcinogenicity of chemical mixtures, such as PFAS and other co-exposed chemicals, and to elucidate their mechanisms.

The AI-CPTM approach not only improved the accuracy of toxicity predictions but also provided deeper insights into the mechanisms underlying chemical toxicity. The method demonstrated the ability to predict the toxic effects of complex mixtures, including those involving PFAS, which are emerging contaminants of concern. Combining machine learning and pathophysiological modeling allowed us to include and analyze chemical interactions and their dose-dependent effects. This integrative approach represents a significant advancement in environmental toxicology, offering a comprehensive tool for assessing the risks associated with chemical exposures. Our study highlighted machine-learning models’ limitations and potential biases, emphasizing the need for ongoing refinement and validation. While AI-CPTM showed marked improvements in predictive accuracy, further research is necessary to ensure its generalizability across different chemical contexts. Future studies should focus on expanding the dataset, incorporating human-cell model validations, and exploring the effects of more complex multi-mixture scenarios. Addressing these challenges will be our priority for broadening the applicability of AI-CPTM and enhancing its utility in regulatory and environmental safety assessments. The continued development and refinement of AI-CPTM is our priority to improve our ability to predict and mitigate the risks posed by hazardous chemical mixtures, ultimately contributing to better environmental and public health outcomes.

## Figures and Tables

**Table 1:** Eleven toxic chemicals that were used to form binary combinations. These combinations are employed by the AI-CPTM model to predict the toxicity of chemical mixtures.

Chemicals	CASRN
Pyraclostrobin	175013-18-0
Fenpropathrin	39515-41-8
Alpha, alpha’-(1-methylethylenediimino)di-ortho-cresol	94-91-7
Paclobutrazol	76738-62-0
2,4,6-Tribromophenol	118-79-6
Pyridaben	96489-71-3
Butachlor	23184-66-9
Tetramethrin	7696-12-0
Dicyclohexyl Phthalate (DCHP)	84-61-7
Perfluorooctanoic acid (PFOA)	335-67-1
Perfluorooctane sulfonic acid (PFOS)	1763-23-1

## Data Availability

All the data supporting reported results can be found in the Supplementary Materials.

## Supplementary Materials

The following supporting information can be downloaded at: https://www.mdpi.com/article/10.3390/toxics12070481/s1, Supplementary Material S1: Supplementary Table S1. Literature collected 981 experimental binary chemical mixtures with citations; Supplementary Table S1 all references [38,39,56,57,58,59,60,61,62,63,64,65,66,67,68,69,70,71,72,73,74,75,76,77,78,79,80,81,82,83,84,85,86,87,88,89,90,91,92,93,94,95,96,97,98,99,100,101,102,103,104,105,106,107,108,109,110,111,112,113]. References cited for supporting the Supplementary Table S1. Supplementary Table S2. Representative set of predicted range of concentration of the chemicals A & B that result in the median effect caused by the mixture for the 160 binary mixtures combinations in the validation set. The values are calculated from predicted pEC50. pA and pB: mass fraction of chemicals A and B; Supplementary Equation (S1). Equations to calculate the evaluation metrics for binary & multiclass classification. Supplementary Equation (S2). Equations to calculate the evaluation metrics for regression models. Supplementary Material S2; The toxicity predictions for binary mixtures of ten chemicals, as detailed in Table 2, using four machine-learning methods: hybrid neural network (HNN), random forest (RF), bagging, and AdaBoost. The table also includes the concentrations of the components in each binary mixture. The table includes the phenotype scores of individual components chemicals in a mixture and their average phenotype score. The last two columns provide experimental toxicity and chemical interaction effects that support a reference to evaluate the accuracy of the machine-learning models.
