# Supplementary material for: A Machine-Learning-Driven Pathophysiology-Based New Approach Method for the Dose-Dependent Assessment of Hazardous Chemical Mixtures and Experimental Validations
Source: Toxics. 2024 Jun 30;12(7):481. doi: 10.3390/toxics12070481 (PMC11281031; doi:10.3390/toxics12070481)
Supplement: Supplementary file 1 [file toxics-12-00481-s001.zip › Supplementary_Material S1.pdf]

Supplementary Material S1

Table S1. Literature collected 981 experimental binary chemical mixtures with citations.

	Species	No of Chemicals	No of data	Sources
Bacteria	*Photobacterium phosphoreum*	8	74	Lin et al, 2002[1]
(805)		8	66	Lin et al, 2003a[2]
		19	29	Lin et al, 2004[3]
		15	14	Ruilian et al, 2010[4]
		8	7	Zou et al, 2012[5]
		12	176	Tian et al, 2013[6]
		5	10	Lin et al, 2003b[7]
		9	15	Wang et al, 2006[8]
		18	46	Yao et al, 2013[9]
		11	23	Wang et al, 2011[10]
	Vibrio qinghaiensis	3	3	Ding et al, 2017[11]
		4	15	Liu et al, 2013[12]
		3	15	Liu et al, 2015[13]
	Escherichia coli	28	90	Long et al, 2016[14]
		5	4	Richter & Escher, 2005[15]
		6	7	Wang et al, 2018a[16]
		24	108	Wang et al, 2018b[17]
	Aliivibrio fischeri	8	14	Wang et al, 2016b[18]
		5	25	Qin et al, 2018[19]
		3	3	Rosal et al, 2010[20]
		3	3	Rodea-Palomares et al, 2010[21]
	Anabaena	8	11	Rodea-Palomares et al, 2012[22]
	E. coli & Vibrio fischeri	22	47	Wang et al, 2016a[23]
Fish	*Onchorhynchus mykiss*	2	1	Howe et al, 1998[24]
(34)	*Pimephales promelas*	2	1	Denton et al, 2003[25]
		2	1	Belden and Lydy, 2006[26]
	Danio rerio	3	2	Schmidt et al, 2016[27]
		16	10	Coors and Frische, 2011[28]
		2	1	Wang et al, 2018c[29]
		2	1	Wu et al, 2018[30]
		2	1	Zhang et al, 2017[31]
		2	4	Ding et al, 2013[32]
		4	6	Wang et al, 2018d[33]
	Cyprinus carpio	4	5	Wang et al, 2015b[34]
	Electrophorus electricus L.	2	1	Froment et al, 2016[35]
Sea-urchin (3)	*P lividus*	3	3	Bellas, 2008[36]
Insect	*Chironomus tentans*	5	4	Anderson & Zhu, 2004[37]
(26)		9	14	Schuler et al, 2005[38]
		5	4	Belden and Lydy, 2000[39]
		3	2	Jin-Clark et al, 2002[40]

	Chironomus riparius	3	2	Pérez et al, 2013[41]
Earthworm	*Eisenia fetida*	3	3	Chen et al, 2014[42]
(7)		3	3	Chen et al, 2014b[43]
		2	1	Wang et al, 2015a[44]
Crustacea	*Tigriopus brevicornis*	3	2	Forget et al, 1999[45]
(34)	*Ceriodaphnia dubia*	3	3	Woods et al, 2002[46]
		4	6	Henry and Black, 2007[47]
		4	4	Choung et al, 2011[48]
	Hyalella azteca	11	10	Trimble and Lydy, 2006[49]
	Daphnia magna	6	4	Bona et al, 2014[50]
		2	3	Puckowski et al, 2017[51]
		3	2	Schell et al, 2018[52]
Cell line	*Fish*	3	3	Bain & Kumar, 2014[53]
(28)	*Human*	5	10	Christen et al, 2014[54]
	Human	4	2	Takakura et al, 2013[55]
	Human	2	1	Savary et al, 2014[56]
		6	12	Scelfo et al, 2012[57]
House fly		7	10	Arora et al, 2017[58]
(44)		5	10	Arora and Kumar, 2015[59]
		7	24	Khan et al, 2013[60]

Table S2. Representative set of predicted range of concentration of the chemicals A & B that result in the median effect caused by the mixture for the 160 binary mixtures combinations in the validation set. The values are calculated from predicted pEC_{50}. p_A and p_B: mass fraction of chemicals A and B.

	CAS of A & B	p_A	p_B	pEC_{50}		EC_{50}	Conc. Range for A		Conc. Range for B	
				True	*Pred*	*Pred*	*lower*	*upper*	*lower*	*upper*
1	68-35-9.61336-70-7	0.972	0.028	2.217	2.061	0.0087	0.00675	0.01013	0.00019	0.00029
2	127-69-5.7169-34-8	0.005	0.995	-1.939	-2.050	112.1642	0.44693	0.67040	89.28440	133.92660
3	120-83-2.95-76-1	0.422	0.578	-1.041	-1.136	13.6760	4.61872	6.92809	6.32211	9.48317
4	127-69-5.3188-00-9	0.074	0.926	-2.971	-3.195	1566.9437	92.73713	139.10569	1160.81783	1741.22675
5	106-37-6.56961-77-4	0.481	0.519	-0.833	-0.681	4.7999	1.84884	2.77327	1.99111	2.98667
6	108-86-1.106-39-8	0.797	0.203	-1.122	-1.143	13.9097	8.86842	13.30263	2.25933	3.38899
7	64-75-5.22122-36-7	0.000	1.000	-4.412	-3.890	7763.3265	0.00594	0.00890	6210.65523	9315.98285
8	108-95-2.3233-58-7	0.830	0.170	-2.332	-1.770	58.9379	39.14102	58.71152	8.00931	12.01397
9	57-68-1.497-23-4	0.000	1.000	-3.985	-3.916	8248.6540	0.17757	0.26635	6598.74566	9898.11848
10	109-77-3.104-88-1	0.873	0.127	-1.882	-1.920	83.1732	58.08125	87.12187	8.45727	12.68591
11	144-83-2.23356-96-9	0.016	0.984	-2.929	-3.091	1233.3673	15.45489	23.18233	971.23897	1456.85846
12	127-69-5.7240-38-2	0.882	0.118	1.522	1.385	0.0412	0.02906	0.04359	0.00390	0.00585
13	109-77-3.100-52-7	0.938	0.062	-1.842	-2.069	117.1558	87.90722	131.86083	5.81741	8.72612
14	109-77-3.111-71-7	0.896	0.104	-1.870	-1.975	94.3324	67.64641	101.46962	7.81951	11.72926
15	307-55-1.67905-19-5	0.476	0.524	-0.928	-0.869	7.3919	2.81237	4.21856	3.10118	4.65178
16	68-35-9.132-98-9	0.997	0.003	2.811	2.440	0.0036	0.00289	0.00434	0.00001	0.00001
17	68-35-9.102280-35-3	0.969	0.031	2.539	2.321	0.0048	0.00370	0.00555	0.00012	0.00018
18	2447-57-6.1125-99-1	0.001	0.999	-3.210	-3.181	1516.7129	0.70123	1.05184	1212.66906	1819.00359
19	120-83-2.95-76-1	0.268	0.732	-0.770	-1.074	11.8459	2.53550	3.80326	6.94120	10.41180
20	109-77-3.105-07-7	0.004	0.996	-1.719	-1.550	35.4783	0.10087	0.15130	28.28177	42.42265
21	108-90-7.106-39-8	0.623	0.377	-1.045	-1.126	13.3706	6.66465	9.99698	4.03180	6.04770

22	109-77-3.123-38-6	0.223	0.777	-2.167	-2.181	151.7760	27.07005	40.60508	94.35077	141.52616
23	109-77-3.110-62-3	0.039	0.961	-1.712	-1.751	56.3490	1.74430	2.61645	43.33486	65.00229
24	100-25-4.528-29-0	0.523	0.477	0.484	-0.817	6.5657	2.74713	4.12069	2.50541	3.75811
25	109-77-3.110-62-3	0.287	0.713	-1.931	-1.832	67.9145	15.59294	23.38941	38.73863	58.10795
26	109-77-3.123-72-8	0.044	0.956	-1.656	-1.882	76.2556	2.67821	4.01732	58.32627	87.48940
27	109-77-3.104-88-1	0.986	0.014	-2.205	-2.143	139.1148	109.70705	164.56058	1.58478	2.37717
28	56-75-7.330-55-2	0.767	0.233	-1.870	-1.848	70.5025	43.24424	64.86635	13.15775	19.73663
29	109-77-3.455-19-6	0.346	0.654	-1.216	-1.256	18.0230	4.99219	7.48828	9.42619	14.13929
30	555-16-8.107-02-8	0.980	0.020	-0.625	-0.966	9.2565	7.25727	10.88591	0.14796	0.22193
31	94-13-3.94-75-7	0.761	0.239	-0.435	-1.443	27.7093	16.86991	25.30487	5.29752	7.94628
32	109-77-3.100-52-7	1.000	0.000	-2.248	-2.223	166.9532	133.55975	200.33962	0.00283	0.00424
33	108-90-7.108-86-1	0.457	0.543	-1.394	-1.335	21.6068	7.90267	11.85400	9.38280	14.07420
34	108-86-1.87-61-6	0.779	0.221	-1.019	-1.092	12.3481	7.69149	11.53723	2.18699	3.28048
35	1173-88-2.149-87-1	0.000	1.000	-3.751	-3.807	6408.5732	0.04322	0.06482	5126.81533	7690.22300
36	109-77-3.105-07-7	0.060	0.940	-1.737	-1.527	33.6446	1.61156	2.41734	25.30409	37.95613
37	64-72-2.149-87-1	0.000	1.000	-3.831	-3.804	6369.5394	0.00406	0.00609	5095.62743	7643.44114
38	2447-57-6.23356-96-9	0.007	0.993	-3.137	-3.114	1300.1552	7.25944	10.88915	1032.86474	1549.29711
39	109-77-3.100-52-7	0.005	0.995	-1.552	-1.460	28.8240	0.10838	0.16257	22.95086	34.42629
40	100-10-7.140-29-4	0.079	0.921	-0.576	-1.166	14.6610	0.92979	1.39469	10.79898	16.19847
41	723-46-6.68832-13-3	0.122	0.878	-3.058	-2.978	950.0032	93.03995	139.55993	666.96260	1000.44390
42	109-77-3.623-27-8	0.338	0.662	-1.223	-1.266	18.4671	4.98751	7.48126	9.78620	14.67931
43	56961-77-4.108-95-2	0.044	0.956	-1.565	-1.735	54.2649	1.89864	2.84796	41.51331	62.26996
44	2447-57-6.7169-34-8	0.024	0.976	-2.224	-2.082	120.7013	2.33587	3.50381	94.22518	141.33777
45	68-35-9.132-98-9	0.939	0.061	2.262	1.979	0.0105	0.00789	0.01184	0.00051	0.00076
46	108-95-2.95-76-1	0.838	0.162	-1.464	-1.793	62.1144	41.63128	62.44692	8.06028	12.09042
47	109-77-3.105-07-7	0.999	0.001	-2.269	-2.220	165.9321	132.67921	199.01882	0.06643	0.09965
48	62-53-3.95-76-1	0.959	0.041	-2.267	-2.486	306.1623	234.87599	352.31399	10.05388	15.08081
49	109-77-3.111-71-7	0.609	0.391	-1.444	-1.630	42.6439	20.77417	31.16125	13.34092	20.01138
50	108-86-1.120-83-2	0.877	0.123	-1.428	-1.287	19.3471	13.57642	20.36463	1.90124	2.85186
51	78-97-7.927-74-2	0.172	0.828	-3.347	-2.851	709.3204	97.33870	146.00805	470.11759	705.17638
52	723-46-6.7169-34-8	0.165	0.835	-1.483	-1.660	45.6617	6.01840	9.02760	30.51098	45.76647
53	80-32-0.2799-21-5	0.166	0.834	-2.593	-2.997	993.9849	132.33013	198.49520	662.85775	994.28663
54	144-83-2.108-29-2	0.015	0.985	-3.874	-3.838	6893.5594	81.51912	122.27869	5433.32840	8149.99260
55	108-95-2.95-76-1	0.954	0.046	-1.612	-1.817	65.5414	50.01241	75.01861	2.42074	3.63111
56	109-77-3.123-38-6	0.900	0.100	-2.254	-2.179	150.8752	108.62707	162.94060	12.07309	18.10963
57	109-77-3.455-19-6	1.000	0.000	-2.269	-2.155	142.9251	114.31899	171.47848	0.02108	0.03162
58	42017-89-0.25812-30-0	0.066	0.934	-0.993	-1.563	36.5352	1.92378	2.88566	27.30435	40.95652
59	71-43-2.87-61-6	0.990	0.010	-2.055	-1.919	82.9718	65.74241	98.61361	0.63507	0.95260
60	108-42-9.95-76-1	0.771	0.229	-1.277	-1.335	21.6364	13.33817	20.00726	3.97095	5.95642
61	56961-77-4.62-53-3	0.020	0.980	-2.414	-2.376	237.4917	3.76611	5.64917	186.22728	279.34092
62	585-79-5.62-53-3	0.027	0.973	-2.375	-2.416	260.8310	5.67233	8.50849	202.99247	304.48870
63	1220-83-3.64485-93-4	0.270	0.730	0.117	0.372	0.4248	0.09187	0.13780	0.24801	0.37201
64	109-77-3.105-07-7	1.000	0.000	-2.270	-2.207	161.2420	128.95748	193.43622	0.03616	0.05424
65	132-98-9.149-87-1	0.000	1.000	-3.761	-3.802	6331.8400	0.00505	0.00757	5065.46697	7598.20045
66	108-95-2.123-30-8	0.754	0.246	-2.179	-1.977	94.7409	57.12432	85.68648	18.66842	28.00263
67	1763-23-1.94-13-3	0.578	0.422	-1.725	-1.079	11.9981	5.54464	8.31696	4.05382	6.08072
68	109-77-3.104-88-1	0.957	0.043	-2.097	-2.093	123.8334	94.80596	142.20895	4.26074	6.39111
69	109-77-3.123-72-8	0.125	0.875	-1.738	-1.910	81.2635	8.15799	12.23698	56.85284	85.27927
70	106-39-8.106-37-6	0.275	0.725	-0.856	-0.917	8.2511	1.81311	2.71966	4.78776	7.18164
71	106-39-8.56961-77-4	0.584	0.416	-0.760	-0.695	4.9558	2.31718	3.47578	1.64742	2.47114
72	375-85-9.376-06-7	0.787	0.213	-0.969	-1.110	12.8701	8.10228	12.15342	2.19382	3.29073
73	375-22-4.307-24-4	0.450	0.550	-1.164	-1.174	14.9252	5.37742	8.06613	6.56274	9.84411
74	314-40-9.2439-10-3	0.996	0.004	-1.947	-1.692	49.2414	39.21976	58.82964	0.17339	0.26009
75	59-40-5.149-87-1	0.000	1.000	-3.911	-3.802	6335.4657	0.16274	0.24410	5068.20981	7602.31472
76	108-95-2.95-57-8	0.812	0.188	-1.866	-1.820	66.1041	42.92565	64.38847	9.95764	14.93645

77	80-35-3.616-45-5	0.000	1.000	-3.170	-3.220	1660.8397	0.15525	0.23288	1328.51653	1992.77479
78	120-83-2.95-76-1	0.154	0.846	-0.980	-1.059	11.4569	1.41548	2.12322	7.75006	11.62509
79	109-77-3.623-27-8	0.998	0.002	-2.257	-2.195	156.7593	125.16761	187.75142	0.23984	0.35976
80	108-86-1.106-46-7	0.733	0.267	-1.208	-1.153	14.2325	8.34608	12.51912	3.03995	4.55993
81	144-83-2.1125-99-1	0.000	1.000	-2.980	-3.159	1442.6575	0.21836	0.32754	1153.90768	1730.86152
82	651-06-9.616-45-5	0.000	1.000	-3.180	-3.218	1652.9618	0.23930	0.35895	1322.13010	1983.19516
83	64-75-5.6981-18-6	0.362	0.638	1.218	0.829	0.1481	0.04289	0.06433	0.07560	0.11340
84	71-36-3.71-41-0	0.716	0.284	-3.208	-3.027	1063.9338	609.07681	913.61521	242.07023	363.10535
85	108-95-2.62-53-3	0.307	0.693	-2.790	-2.346	221.9507	54.44062	81.66092	123.11993	184.67989
86	555-16-8.109-75-1	0.013	0.987	-2.470	-2.403	252.7648	2.70777	4.06166	199.50405	299.25607
87	106-39-8.106-37-6	0.602	0.398	-0.866	-0.947	8.8432	4.26139	6.39209	2.81320	4.21980
88	64-75-5.330-55-2	0.771	0.229	-1.338	-0.770	5.8821	3.62969	5.44453	1.07602	1.61402
89	109-77-3.110-62-3	0.004	0.996	-1.671	-1.752	56.4600	0.18108	0.27162	44.98691	67.48037
90	123-08-0.109-75-1	0.006	0.994	-2.468	-2.431	269.9105	1.29370	1.94055	214.63468	321.95203
91	555-16-8.107-16-4	0.117	0.883	-1.529	-1.600	39.8067	3.73219	5.59829	28.11319	42.16979
92	67-45-8.50-07-7	0.986	0.014	0.678	-1.119	13.1554	10.37521	15.56281	0.14913	0.22370
93	109-77-3.111-71-7	0.999	0.001	-1.550	-2.078	119.5659	95.59134	143.38701	0.06139	0.09208
94	723-46-6.7169-34-8	0.798	0.202	-0.662	-0.344	2.2088	1.40968	2.11453	0.35733	0.53599
95	106-39-8.106-37-6	0.431	0.569	-0.841	-0.931	8.5367	2.94329	4.41493	3.88608	5.82912
96	80-32-0.5355-16-8	0.871	0.129	1.841	1.928	0.0118	0.00822	0.01233	0.00122	0.00183
97	67905-19-5.16517-11-6	0.061	0.939	-1.118	-0.955	9.0219	0.43743	0.65615	6.78006	10.17008
98	111-70-6.111-87-5	0.822	0.178	-1.065	-1.147	14.0212	9.22430	13.83645	1.99263	2.98895
99	67905-19-5.16517-11-6	0.866	0.134	-0.917	-0.872	7.4546	5.16339	7.74509	0.80031	1.20046
100	100-10-7.100-47-0	0.017	0.983	-1.236	-1.350	22.3716	0.30598	0.45896	17.59132	26.38698
101	64-75-5.62-73-7	0.069	0.931	-2.011	-2.049	111.9675	6.19264	9.28896	83.38133	125.07199
102	109-77-3.555-16-8	1.000	0.000	-2.268	-2.183	152.3449	121.84711	182.77067	0.02883	0.04325
103	67905-19-5.16517-11-6	0.392	0.608	-0.971	-0.896	7.8708	2.46929	3.70394	3.82731	5.74097
104	57-68-1.149-87-1	0.000	1.000	-3.851	-3.859	7230.2143	0.31319	0.46978	5783.85823	8675.78734
105	138261-41-3.23103-98-2	0.363	0.637	-2.008	-1.929	84.9116	24.66964	37.00446	43.25961	64.88941
106	109-77-3.123-38-6	0.994	0.006	-2.269	-2.181	151.8726	120.74652	181.11978	0.75152	1.12729
107	109-77-3.455-19-6	0.990	0.010	-2.203	-2.103	126.8382	100.41253	150.61879	1.05802	1.58703
108	723-46-6.102280-35-3	0.948	0.052	2.522	2.369	0.0043	0.00325	0.00487	0.00018	0.00026
109	64-75-5.616-45-5	0.000	1.000	-3.290	-3.252	1786.0511	0.00509	0.00764	1428.83576	2143.25364
110	109-77-3.75-07-0	0.088	0.912	-2.259	-2.261	182.1901	12.86651	19.29976	132.88558	199.32838
111	123-72-8.75-86-5	0.660	0.340	-1.491	-1.805	63.8476	33.71028	50.56542	17.36781	26.05172
112	64-72-2.616-45-5	0.000	1.000	-3.320	-3.285	1925.8071	0.00240	0.00360	1540.64326	2310.96489
113	108-95-2.51-28-5	0.905	0.095	-2.024	-1.776	59.7102	43.22693	64.84040	4.54125	6.81187
114	62-53-3.95-76-1	0.979	0.021	-2.473	-2.368	233.4889	182.87709	274.31563	3.91403	5.87105
115	98-95-3.95-76-1	0.879	0.121	-1.583	-1.426	26.6951	18.76959	28.15439	2.58647	3.87971
116	109-77-3.455-19-6	0.968	0.032	-2.093	-2.067	116.7849	90.46246	135.69369	2.96545	4.44818
117	80-32-0.61336-70-7	0.973	0.027	2.122	2.049	0.0089	0.00694	0.01042	0.00019	0.00029
118	2447-57-6.91296-87-6	0.964	0.036	2.034	1.972	0.0107	0.00823	0.01235	0.00031	0.00046
119	2447-57-6.738-70-5	0.992	0.008	2.748	2.101	0.0079	0.00628	0.00942	0.00005	0.00008
120	109-77-3.555-16-8	0.295	0.705	-1.042	-1.111	12.9025	3.04901	4.57352	7.27301	10.90951
121	111-70-6.111-87-5	0.536	0.464	-0.981	-1.067	11.6646	5.00604	7.50907	4.32562	6.48842
122	108-95-2.95-48-7	0.788	0.212	-2.316	-1.968	92.9652	58.59016	87.88524	15.78198	23.67297
123	2447-57-6.61336-70-7	0.986	0.014	2.147	1.983	0.0104	0.00821	0.01231	0.00011	0.00017
124	109-77-3.100-52-7	0.825	0.175	-1.644	-1.853	71.2922	47.06667	70.60000	9.96710	14.95065
125	2447-57-6.149418-41-7	0.000	1.000	-4.124	-4.063	11562.3412	0.22614	0.33921	9249.64679	13874.47018
126	80-35-3.6981-18-6	0.971	0.029	2.263	2.361	0.0044	0.00338	0.00507	0.00010	0.00015
127	109-77-3.123-38-6	1.000	0.000	-2.270	-2.176	149.8022	119.82846	179.74269	0.01332	0.01998
128	108-90-7.108-95-2	0.298	0.702	-1.795	-1.834	68.2371	16.29060	24.43590	38.29906	57.44858
129	80-32-0.6981-18-6	0.943	0.057	2.307	2.158	0.0070	0.00524	0.00786	0.00032	0.00048
130	106-46-7.87-61-6	0.719	0.281	-0.811	-0.858	7.2083	4.14769	6.22153	1.61893	2.42839
131	314-40-9.2439-10-3	0.978	0.022	-1.638	-1.658	45.4912	35.60112	53.40168	0.79181	1.18772

132	108-90-7.106-39-8	0.768	0.232	-1.125	-1.202	15.9155	9.77554	14.66331	2.95687	4.43530
133	51235-04-2.2439-10-3	0.999	0.001	-2.502	-2.206	160.6275	128.35562	192.53343	0.14637	0.21956
134	109-77-3.123-38-6	0.616	0.384	-2.214	-2.154	142.5593	70.29557	105.44336	43.75184	65.62775
135	109-77-3.104-88-1	0.794	0.206	-1.747	-1.784	60.7526	38.60787	57.91180	9.99419	14.99129
136	56-75-7.330-55-2	0.988	0.012	-2.498	-1.860	72.4964	57.27916	85.91875	0.71797	1.07696
137	106-46-7.106-39-8	0.417	0.583	-0.840	-0.981	9.5662	3.19022	4.78533	4.46271	6.69407
138	71-43-2.56961-77-4	0.953	0.047	-1.786	-1.778	59.9360	45.69913	68.54869	2.24964	3.37447
139	109-77-3.455-19-6	0.994	0.006	-2.232	-2.132	135.6342	107.87090	161.80635	0.63650	0.95475
140	59-40-5.616-45-5	0.000	1.000	-3.160	-3.178	1506.9818	0.07565	0.11348	1205.50976	1808.26464
141	71-43-2.108-90-7	0.814	0.186	-1.818	-1.807	64.1409	41.75459	62.63188	9.55815	14.33723
142	100-10-7.107-16-4	0.010	0.990	-1.479	-1.691	49.0961	0.38665	0.57998	38.89025	58.33537
143	59-40-5.86483-48-9	0.936	0.064	1.887	2.050	0.0089	0.00667	0.01000	0.00046	0.00069
144	80-32-0.616-45-5	0.000	1.000	-3.100	-3.211	1624.1873	0.04991	0.07486	1299.29993	1948.94990
145	109-77-3.111-71-7	0.999	0.001	-2.263	-2.098	125.4045	100.20866	150.31298	0.11492	0.17237
146	1220-83-3.70458-96-7	0.785	0.215	1.191	1.069	0.0853	0.05358	0.08036	0.01464	0.02196
147	109-77-3.100-52-7	0.979	0.021	-1.919	-2.124	133.1221	104.28919	156.43378	2.20849	3.31273
148	577-11-7.3380-34-5	0.999	0.001	-0.836	-1.321	20.9290	16.72751	25.09126	0.01567	0.02350
149	67905-19-5.16517-11-6	0.114	0.886	-1.075	-0.953	8.9713	0.82025	1.23038	6.35681	9.53522
150	307-55-1.376-06-7	0.599	0.401	-0.773	-0.877	7.5396	3.61361	5.42041	2.41808	3.62712
151	108-95-2.95-76-1	0.912	0.088	-1.830	-1.796	62.4843	45.57545	68.36317	4.41196	6.61793
152	106-39-8.87-61-6	0.642	0.358	-0.624	-0.760	5.7599	2.95749	4.43623	1.65043	2.47564
153	818-61-1.106-54-7	0.963	0.037	-1.898	-1.740	55.0044	42.37144	63.55715	1.63210	2.44815
154	109-77-3.100-52-7	1.000	0.000	-2.238	-2.198	157.7753	126.19350	189.29025	0.02672	0.04009
155	127-69-5.7169-34-8	0.334	0.666	-1.206	-1.394	24.7770	6.61223	9.91834	13.20939	19.81408
156	59-40-5.70458-96-7	0.753	0.247	1.216	1.351	0.0446	0.02683	0.04024	0.00882	0.01322
157	108-90-7.106-37-6	0.556	0.444	-1.156	-1.105	12.7225	5.65841	8.48762	4.51955	6.77932
158	109-77-3.111-71-7	0.996	0.004	-2.088	-2.169	147.6987	117.73892	176.60839	0.42006	0.63009
159	106-46-7.106-37-6	0.520	0.480	-0.914	-0.948	8.8616	3.68566	5.52849	3.40363	5.10545
160	106-46-7.106-39-8	0.588	0.412	-0.851	-0.990	9.7689	4.59863	6.89795	3.21645	4.82468

Equation S1. Equations to calculate the evaluation metrics for binary & multiclass classification.

$$Accuracy = \frac{TP + TN}{TP + TN + FN + FP} \times 100$$

$$Sensitivity(truepositiverate) = \frac{TP}{TP + FN} \times 100$$

$$Specificity(truenegativerate) = \frac{TN}{TN + FP} \times 100$$

Where, TP = True Positive, TN = True Negative, FP = False Positive, and FN = False Negative.

Equation S2. Equations to calculate the evaluation metrics for regression models.

$$R^2 = \frac{ESS}{TSS} = \frac{\sum_{i=1}^{n} (\hat{y}_i - \bar{y})^2}{\sum_{i=1}^{n} (y_i - \bar{y})^2}$$

$$MSE = \frac{\sum_{i=1}^{n} |\hat{y}_i - y_i|^2}{n}$$

$$MAE = \frac{\sum_{i=1}^{n} |\hat{y}_i - y_i|}{n}$$

Where, ESS is explained sum of squares and TSS is the total sum of squares, $\hat{y}_i$ is the predicted value of the ith dependent variable, y_i is the ith observed dependent variable, and $\bar{y}$ is the mean of the observed data.

Supplementary Table S1 Ref. 1-60. References cited for supporting the Supplementary Table S1.

(1) Lin, Z.; Yu, H.; Wei, D.; Wang, G.; Feng, J.; Wang, L. Prediction of Mixture Toxicity with Its Total Hydrophobicity. *Chemosphere* **2002**, *46* (2), 305–310. https://doi.org/10.1016/s0045-6535(01)00083-2.

(2) Lin, Z.; Zhong, P.; Yin, K.; Wang, L.; Yu, H. Quantification of Joint Effect for Hydrogen Bond and Development of QSARs for Predicting Mixture Toxicity. *Chemosphere* **2003**, *52* (7), 1199–1208. https://doi.org/10.1016/S0045-6535(03)00329-1.

(3) Lin, Z.; Du, J.; Yin, K.; Wang, L.; Yu, H. Mechanism of Concentration Addition Toxicity: They Are Different for Nonpolar Narcotic Chemicals, Polar Narcotic Chemicals and Reactive Chemicals. *Chemosphere* **2004**, *54* (11), 1691–1701. https://doi.org/10.1016/j.chemosphere.2003.09.031.

(4) Ruilian, Y.; Jiaqing, X.; Junjun, L. Evaluation on the Binary Joint Toxicity of Phenol with Substituted Phenols to Photobacterium Phosphoreum Using Four Evaluating Methods. In *2010 International Conference on Digital Manufacturing Automation*; 2010; Vol. 1, pp 674–677. https://doi.org/10.1109/ICDMA.2010.341.

(5) Zou, X.; Lin, Z.; Deng, Z.; Yin, D.; Zhang, Y. The Joint Effects of Sulfonamides and Their Potentiator on Photobacterium Phosphoreum: Differences between the Acute and Chronic Mixture Toxicity Mechanisms. *Chemosphere* **2012**, *86* (1), 30–35. https://doi.org/10.1016/j.chemosphere.2011.08.046.

(6) Tian, D.; Lin, Z.; Zhou, X.; Yin, D. The Underlying Toxicological Mechanism of Chemical Mixtures: A Case Study on Mixture Toxicity of Cyanogenic Toxicants and Aldehydes to Photobacterium Phosphoreum. *Toxicol. Appl. Pharmacol.* **2013**, *272* (2), 551–558. https://doi.org/10.1016/j.taap.2013.06.015.

(7) Lin, Z.; Shi, P.; Gao, S.; Wang, L.; Yu, H. Use of Partition Coefficients to Predict Mixture Toxicity. *Water Res.* **2003**, *37* (9), 2223–2227. https://doi.org/10.1016/S0043-1354(02)00619-X.

(8) Wang, B.; Yu, G.; Zhang, Z.; Hu, H.; Wang, L. Quantitative Structure-Activity Relationship and Prediction of Mixture Toxicity of Alkanols. *Chin. Sci. Bull.* **2006**, *51* (22), 2717–2723. https://doi.org/10.1007/s11434-006-2168-z.

(9) Yao, Z.; Lin, Z.; Wang, T.; Tian, D.; Zou, X.; Gao, Y.; Yin, D. Using Molecular Docking-Based Binding Energy to Predict Toxicity of Binary Mixture with Different Binding Sites. *Chemosphere* **2013**, *92* (9), 1169–1176. https://doi.org/10.1016/j.chemosphere.2013.01.081.

(10) Wang, T.; Lin, Z.; Yin, D.; Tian, D.; Zhang, Y.; Kong, D. Hydrophobicity-Dependent QSARs to Predict the Toxicity of Perfluorinated Carboxylic Acids and Their Mixtures. *Environ. Toxicol. Pharmacol.* **2011**, *32* (2), 259–265. https://doi.org/10.1016/j.etap.2011.05.011.

(11) Ding, K.; Lu, L.; Wang, J.; Wang, J.; Zhou, M.; Zheng, C.; Liu, J.; Zhang, C.; Zhuang, S. In Vitro and in Silico Investigations of the Binary-Mixture Toxicity of Phthalate Esters and Cadmium (II) to Vibrio Qinghaiensis Sp.-Q67. *Sci. Total Environ.* **2017**, *580*, 1078–1084. https://doi.org/10.1016/j.scitotenv.2016.12.062.

(12) Liu, S.-S.; Wang, C.-L.; Zhang, J.; Zhu, X.-W.; Li, W.-Y. Combined Toxicity of Pesticide Mixtures on Green Algae and Photobacteria. *Ecotoxicol. Environ. Saf.* **2013**, *95*, 98–103. https://doi.org/10.1016/j.ecoenv.2013.05.018.

(13) Liu, L.; Liu, S.-S.; Yu, M.; Chen, F. Application of the Combination Index Integrated with Confidence Intervals to Study the Toxicological Interactions of Antibiotics and Pesticides in Vibrio Qinghaiensis Sp.-Q67. *Environ. Toxicol. Pharmacol.* **2015**, *39* (1), 447–456. https://doi.org/10.1016/j.etap.2014.12.013.

(14) Long, X.; Wang, D.; Lin, Z.; Qin, M.; Song, C.; Liu, Y. The Mixture Toxicity of Environmental Contaminants Containing Sulfonamides and Other Antibiotics in Escherichia Coli: Differences in Both the Special Target Proteins of Individual Chemicals and Their Effective Combined Concentration. *Chemosphere* **2016**, *158*, 193–203. https://doi.org/10.1016/j.chemosphere.2016.05.048.

(15) Richter, M.; Escher, B. I. Mixture Toxicity of Reactive Chemicals by Using Two Bacterial Growth Assays as Indicators of Protein and DNA Damage. *Environ. Sci. Technol.* **2005**, *39* (22), 8753–8761. https://doi.org/10.1021/es050758o.

(16) Wang, D.; Wu, X.; Lin, Z.; Ding, Y. A Comparative Study on the Binary and Ternary Mixture Toxicity of Antibiotics towards Three Bacteria Based on QSAR Investigation. *Environ. Res.* **2018**, *162*, 127–134. https://doi.org/10.1016/j.envres.2017.12.015.
(17) Wang, D.; Shi, J.; Xiong, Y.; Hu, J.; Lin, Z.; Qiu, Y.; Cheng, J. A QSAR-Based Mechanistic Study on the Combined Toxicity of Antibiotics and Quorum Sensing Inhibitors against Escherichia Coli. *J. Hazard. Mater.* **2018**, *341*, 438–447. https://doi.org/10.1016/j.jhazmat.2017.07.059.
(18) Wang, T.; Liu, Y.; Wang, D.; Lin, Z.; An, Q.; Yin, C.; Liu, Y. The Joint Effects of Sulfonamides and Quorum Sensing Inhibitors on Vibrio Fischeri: Differences between the Acute and Chronic Mixed Toxicity Mechanisms. *J. Hazard. Mater.* **2016**, *310*, 56–67. https://doi.org/10.1016/j.jhazmat.2016.01.061.
(19) Qin, L.-T.; Chen, Y.-H.; Zhang, X.; Mo, L.-Y.; Zeng, H.-H.; Liang, Y.-P. QSAR Prediction of Additive and Non-Additive Mixture Toxicities of Antibiotics and Pesticide. *Chemosphere* **2018**, *198*, 122–129. https://doi.org/10.1016/j.chemosphere.2018.01.142.
(20) Rosal, R.; Rodea-Palomares, I.; Boltes, K.; Fernández-Piñas, F.; Leganés, F.; Petre, A. Ecotoxicological Assessment of Surfactants in the Aquatic Environment: Combined Toxicity of Docusate Sodium with Chlorinated Pollutants. *Chemosphere* **2010**, *81* (2), 288–293. https://doi.org/10.1016/j.chemosphere.2010.05.050.
(21) Rodea-Palomares, I.; Petre, A. L.; Boltes, K.; Leganés, F.; Perdigón-Melón, J. A.; Rosal, R.; Fernández-Piñas, F. Application of the Combination Index (CI)-Isobologram Equation to Study the Toxicological Interactions of Lipid Regulators in Two Aquatic Bioluminescent Organisms. *Water Res.* **2010**, *44* (2), 427–438. https://doi.org/10.1016/j.watres.2009.07.026.
(22) Rodea-Palomares, I.; Leganés, F.; Rosal, R.; Fernández-Piñas, F. Toxicological Interactions of Perfluorooctane Sulfonic Acid (PFOS) and Perfluorooctanoic Acid (PFOA) with Selected Pollutants. *J. Hazard. Mater.* **2012**.
(23) Wang, T.; Wang, D.; Lin, Z.; An, Q.; Yin, C.; Huang, Q. Prediction of Mixture Toxicity from the Hormesis of a Single Chemical: A Case Study of Combinations of Antibiotics and Quorum-Sensing Inhibitors with Gram-Negative Bacteria. *Chemosphere* **2016**, *150*, 159–167. https://doi.org/10.1016/j.chemosphere.2016.02.018.
(24) Howe, G. E.; Gillis, R.; Mowbray, R. C. Effect of Chemical Synergy and Larval Stage on the Toxicity of Atrazine and Alachlor to Amphibian Larvae. *Environ. Toxicol. Chem.* **1998**, *17* (3), 519–525. https://doi.org/10.1002/etc.5620170324.
(25) Denton, D. L.; Wheelock, C. E.; Murray, S. A.; Deanovic, L. A.; Hammock, B. D.; Hinton, D. E. Joint Acute Toxicity of Esfenvalerate and Diazinon to Larval Fathead Minnows (Pimephales Promelas). *Environ. Toxicol. Chem.* **2003**, *22* (2), 336–341.
(26) Belden, J. B.; Lydy, M. J. Joint Toxicity of Chlorpyrifos and Esfenvalerate to Fathead Minnows and Midge Larvae. *Environ. Toxicol. Chem.* **2006**, *25* (2), 623–629. https://doi.org/10.1897/05-370r.1.
(27) Schmidt, S.; Busch, W.; Altenburger, R.; Küster, E. Mixture Toxicity of Water Contaminants-Effect Analysis Using the Zebrafish Embryo Assay (Danio Rerio). *Chemosphere* **2016**, *152*, 503–512. https://doi.org/10.1016/j.chemosphere.2016.03.006.
(28) Coors, A.; Frische, T. Predicting the Aquatic Toxicity of Commercial Pesticide Mixtures. *Environ. Sci. Eur.* **2011**, *23* (1), 22. https://doi.org/10.1186/2190-4715-23-22.
(29) Wang, Y.; Dai, D.; Yu, Y.; Yang, G.; Shen, W.; Wang, Q.; Weng, H.; Zhao, X. Evaluation of Joint Effects of Cyprodinil and Kresoxim-Methyl on Zebrafish, Danio Rerio. *J. Hazard. Mater.* **2018**, *352*, 80–91. https://doi.org/10.1016/j.jhazmat.2018.03.023.
(30) Wu, S.; Li, X.; Liu, X.; Yang, G.; An, X.; Wang, Q.; Wang, Y. Joint Toxic Effects of Triazophos and Imidacloprid on Zebrafish (Danio Rerio). *Environ. Pollut. Barking Essex 1987* **2018**, *235*, 470–481. https://doi.org/10.1016/j.envpol.2017.12.120.
(31) Zhang, J.; Liu, L.; Ren, L.; Feng, W.; Lv, P.; Wu, W.; Yan, Y. The Single and Joint Toxicity Effects of Chlorpyrifos and Beta-Cypermethrin in Zebrafish (Danio Rerio) Early Life Stages. *J. Hazard. Mater.* **2017**, *334*, 121–131. https://doi.org/10.1016/j.jhazmat.2017.03.055.
(32) Ding, G.; Zhang, J.; Chen, Y.; Wang, L.; Wang, M.; Xiong, D.; Sun, Y. Combined Effects of PFOS and PFOA on Zebrafish (Danio Rerio) Embryos. *Arch. Environ. Contam. Toxicol.* **2013**, *64* (4), 668–675. https://doi.org/10.1007/s00244-012-9864-2.
(33) Wang, Y.; Wu, S.; Chen, J.; Zhang, C.; Xu, Z.; Li, G.; Cai, L.; Shen, W.; Wang, Q. Single and Joint Toxicity Assessment of Four Currently Used Pesticides to Zebrafish (Danio Rerio) Using Traditional and

Molecular Endpoints. *Chemosphere* **2018**, *192*, 14–23. https://doi.org/10.1016/j.chemosphere.2017.10.129.
(34) Wang, Y.; Chen, C.; Zhao, X.; Wang, Q.; Qian, Y. Assessing Joint Toxicity of Four Organophosphate and Carbamate Insecticides in Common Carp (Cyprinus Carpio) Using Acetylcholinesterase Activity as an Endpoint. *Pestic. Biochem. Physiol.* **2015**, *122*, 81–85. https://doi.org/10.1016/j.pestbp.2014.12.017.
(35) Froment, J.; Thomas, K. V.; Tollefsen, K. E. Automated High-Throughput in Vitro Screening of the Acetylcholine Esterase Inhibiting Potential of Environmental Samples, Mixtures and Single Compounds. *Ecotoxicol. Environ. Saf.* **2016**, *130*, 74–80. https://doi.org/10.1016/j.ecoenv.2016.04.005.
(36) Bellas, J. Prediction and Assessment of Mixture Toxicity of Compounds in Antifouling Paints Using the Sea-Urchin Embryo-Larval Bioassay. *Aquat. Toxicol. Amst. Neth.* **2008**, *88* (4), 308–315. https://doi.org/10.1016/j.aquatox.2008.05.011.
(37) Anderson, T. D.; Zhu, K. Y. Synergistic and Antagonistic Effects of Atrazine on the Toxicity of Organophosphorodithioate and Organophosphorothioate Insecticides to Chironomus Tentans (Diptera: Chironomidae). *Pestic. Biochem. Physiol.* **2004**, *80* (1), 54–64. https://doi.org/10.1016/j.pestbp.2004.06.003.
(38) Schuler, L. J.; Trimble, A. J.; Belden, J. B.; Lydy, M. J. Joint Toxicity of Triazine Herbicides and Organophosphate Insecticides to the Midge Chironomus Tentans. *Arch. Environ. Contam. Toxicol.* **2005**, *49* (2), 173–177. https://doi.org/10.1007/s00244-004-0224-8.
(39) Belden, J. B.; Lydy, M. J. Impact of Atrazine on Organophosphate Insecticide Toxicity. *Environ. Toxicol. Chem.* **2000**, *19* (9), 2266–2274. https://doi.org/10.1002/etc.5620190917.
(40) Jin-Clark, Y.; Lydy, M. J.; Zhu, K. Y. Effects of Atrazine and Cyanazine on Chlorpyrifos Toxicity in Chironomus Tentans (Diptera: Chironomidae). *Environ. Toxicol. Chem.* **2002**, *21* (3), 598–603. https://doi.org/10.1897/1551-5028(2002)021<0598:eoaaco>2.0.co;2.
(41) Pérez, J.; Monteiro, M. S.; Quintaneiro, C.; Soares, A. M. V. M.; Loureiro, S. Characterization of Cholinesterases in Chironomus Riparius and the Effects of Three Herbicides on Chlorpyrifos Toxicity. *Aquat. Toxicol. Amst. Neth.* **2013**, *144–145*, 296–302. https://doi.org/10.1016/j.aquatox.2013.10.014.
(42) Chen, C.; Wang, Y.; Zhao, X.; Qian, Y.; Wang, Q. Combined Toxicity of Butachlor, Atrazine and λ-Cyhalothrin on the Earthworm Eisenia Fetida by Combination Index (CI)-Isobologram Method. *Chemosphere* **2014**, *112*, 393–401. https://doi.org/10.1016/j.chemosphere.2014.04.070.
(43) Chen, C.; Wang, Y.; Zhao, X.; Wang, Q.; Qian, Y. Comparative and Combined Acute Toxicity of Butachlor, Imidacloprid and Chlorpyrifos on Earthworm, Eisenia Fetida. *Chemosphere* **2014**, *100*, 111–115. https://doi.org/10.1016/j.chemosphere.2013.12.023.
(44) Wang, Y.; Chen, C.; Qian, Y.; Zhao, X.; Wang, Q.; Kong, X. Toxicity of Mixtures of λ-Cyhalothrin, Imidacloprid and Cadmium on the Earthworm Eisenia Fetida by Combination Index (CI)-Isobologram Method. *Ecotoxicol. Environ. Saf.* **2015**, *111*, 242–247. https://doi.org/10.1016/j.ecoenv.2014.10.015.
(45) Forget, J.; Pavillon, J.-F.; Beliaeff, B.; Bocquené, G. Joint action of pollutant combinations (pesticides and metals) on survival (LC50 values) and acetylcholinesterase activity of Tigriopus brevicornis (Copepoda, Harpacticoida). *Environ. Toxicol. Chem.* **1999**, *18* (5), 912–918. https://doi.org/10.1002/etc.5620180514.
(46) Woods, M.; Kumar, A.; Correll, R. Acute Toxicity of Mixtures of Chlorpyrifos, Profenofos, and Endosulfan to Ceriodaphnia Dubia. *Bull. Environ. Contam. Toxicol.* **2002**, *68* (6), 801–808. https://doi.org/10.1007/s00128-002-0026-5.
(47) Henry, T. B.; Black, M. C. Mixture and Single-Substance Acute Toxicity of Selective Serotonin Reuptake Inhibitors in Ceriodaphnia Dubia. *Environ. Toxicol. Chem.* **2007**, *26* (8), 1751–1755. https://doi.org/10.1897/06-265r.1.
(48) Choung, C. B.; Hyne, R. V.; Stevens, M. M.; Hose, G. C. Toxicity of the Insecticide Terbufos, Its Oxidation Metabolites, and the Herbicide Atrazine in Binary Mixtures to Ceriodaphnia Cf Dubia. *Arch. Environ. Contam. Toxicol.* **2011**, *60* (3), 417–425. https://doi.org/10.1007/s00244-010-9560-z.
(49) Trimble, A. J.; Lydy, M. J. Effects of Triazine Herbicides on Organophosphate Insecticide Toxicity in Hyalella Azteca. *Arch. Environ. Contam. Toxicol.* **2006**, *51* (1), 29–34. https://doi.org/10.1007/s00244-005-0176-7.
(50) Dalla Bona, M.; Di Leva, V.; De Liguoro, M. The Sensitivity of Daphnia Magna and Daphnia Curvirostris to 10 Veterinary Antibacterials and to Some of Their Binary Mixtures. *Chemosphere* **2014**, *115*, 67–74. https://doi.org/10.1016/j.chemosphere.2014.02.003.

(51) Puckowski, A.; Stolte, S.; Wagil, M.; Markiewicz, M.; Łukaszewicz, P.; Stepnowski, P.; Białk-Bielińska, A. Mixture Toxicity of Flubendazole and Fenbendazole to Daphnia Magna. *Int. J. Hyg. Environ. Health* **2017**, *220* (3), 575–582. https://doi.org/10.1016/j.ijheh.2017.01.011.
(52) Schell, T.; Goedkoop, W.; Zubrod, J. P.; Feckler, A.; Lüderwald, S.; Schulz, R.; Bundschuh, M. Assessing the Effects of Field-Relevant Pesticide Mixtures for Their Compliance with the Concentration Addition Model – An Experimental Approach with Daphnia Magna. *Sci. Total Environ.* **2018**, *644*, 342–349. https://doi.org/10.1016/j.scitotenv.2018.06.334.
(53) Bain, P. A.; Kumar, A. Cytotoxicity of Binary Mixtures of Human Pharmaceuticals in a Fish Cell Line: Approaches for Non-Monotonic Concentration-Response Relationships. *Chemosphere* **2014**, *108*, 334–342. https://doi.org/10.1016/j.chemosphere.2014.01.077.
(54) Christen, V.; Crettaz, P.; Fent, K. Additive and Synergistic Antiandrogenic Activities of Mixtures of Azol Fungicides and Vinclozolin. *Toxicol. Appl. Pharmacol.* **2014**, *279* (3), 455–466. https://doi.org/10.1016/j.taap.2014.06.025.
(55) Takakura, N.; Sanders, P.; Fessard, V.; Le Hégarat, L. In Vitro Combined Cytotoxic Effects of Pesticide Cocktails Simultaneously Found in the French Diet. *Food Chem. Toxicol.* **2013**, *52*, 153–162. https://doi.org/10.1016/j.fct.2012.11.011.
(56) Savary, C. C.; Jossé, R.; Bruyère, A.; Guillet, F.; Robin, M.-A.; Guillouzo, A. Interactions of Endosulfan and Methoxychlor Involving CYP3A4 and CYP2B6 in Human HepaRG Cells. *Drug Metab. Dispos. Biol. Fate Chem.* **2014**, *42* (8), 1235–1240. https://doi.org/10.1124/dmd.114.057786.
(57) Scelfo, B.; Politi, M.; Reniero, F.; Palosaari, T.; Whelan, M.; Zaldívar, J.-M. Application of Multielectrode Array (MEA) Chips for the Evaluation of Mixtures Neurotoxicity. *Toxicology* **2012**, *299* (2), 172–183. https://doi.org/10.1016/j.tox.2012.05.020.
(58) Arora, S.; Balotra, S.; Pandey, G.; Kumar, A. Binary Combinations of Organophosphorus and Synthetic Pyrethroids Are More Potent Acetylcholinesterase Inhibitors than Organophosphorus and Carbamate Mixtures: An in Vitro Assessment. *Toxicol. Lett.* **2017**, *268*, 8–16. https://doi.org/10.1016/j.toxlet.2016.12.009.
(59) Arora, S.; Kumar, A. Binary Combinations of Organophosphorus Pesticides Exhibit Differential Toxicity under Oxidised and Un-Oxidised Conditions. *Ecotoxicol. Environ. Saf.* **2015**, *115*, 93–100. https://doi.org/10.1016/j.ecoenv.2015.01.003.
(60) Khan, H. A. A.; Akram, W.; Shad, S. A.; Lee, J.-J. Insecticide Mixtures Could Enhance the Toxicity of Insecticides in a Resistant Dairy Population of Musca Domestica L. *PLOS ONE* **2013**, *8* (4), e60929. https://doi.org/10.1371/journal.pone.0060929.
